# Hsa_circ_0026782 Acts as a “Molecular Break” of CREB1‐Mediated Transcription by Promoting Its Phosphorylation at Ser142 That Prevents Keloid Progression

**DOI:** 10.1002/advs.202508647

**Published:** 2025-08-18

**Authors:** Xin‐Cao Zhong, Chun‐Ye Chen, Zi‐Xuan Feng, Yong Wang, Tao Zhang, Ze‐Ming Zhuang, Zhang‐Rui Wu, Yong‐Zhong Du, Jun Chen, Xiao‐Ying Lin, Wei‐Qiang Tan

**Affiliations:** ^1^ Department of Plastic Surgery Sir Run Run Shaw Hospital Zhejiang University School of Medicine 3 East Qingchun Road Hangzhou 310016 China; ^2^ Institute of Pharmaceutics College of Pharmaceutical Sciences Zhejiang University 866 Yu‐Hang‐Tang Road Hangzhou 310058 China; ^3^ MOE Key Laboratory of Biosystems Homeostasis & Protection College of Life Sciences Zhejiang University 866 Yu‐Hang‐Tang Road Hangzhou 310058 China

**Keywords:** CREB1, hsa_circ_0026782, keloid, Ser133, Ser142

## Abstract

Keloids are a form of excessive fibrosis disease characterized by tumor‐like features, which are prone to recurrence. Circular RNAs play a role in various diseases, but its roles in keloids remain unclear. In this study, using high‐throughput RNA sequencing to compare keloids with normal scars, a novel circRNA, hsa_circ_0026782 is identified, whose expression is downregulated in keloids. The hsa_circ_0026782 inhibits the proliferation, migration, invasion, and apoptosis of primary human keloid fibroblasts, blocks the cell cycle in the S phase in vitro, and prevents keloid progression in vivo. Mechanistically, 1–90 nucleotides of hsa_circ_0026782 directly bind to the transcription factor CREB1. This interaction increases the exposure of the basic leucine zipper domain of CREB1 to enhance its dimerization. In addition, this interaction also promotes CREB1 phosphorylation at serine residue 142, inhibits the binding of CREB1 to the promoters or enhancers of its downstream target genes, and ultimately alters the transactivation of CREB1. The findings unveil that the hsa_circ_0026782/CREB1 axis acts as a transcriptional spatiotemporal “molecular break” in the formation of keloids, providing a new target for the therapy of keloids.

## Graphical abstract

Model for the role of hsa_circ_0026782/p‐CREB1 (Ser142) played in keloid. Hsa_circ_0026782 is derived from back‐splicing of ITGA7 exon 4. When hsa_circ_0026782 binds to CREB1, the exposure of the bZIP domain and dimerization of CREB1 are promoted (upper left). Hsa_circ_0026782 increases the phosphorylation of Ser142 without affecting the expression, chromatin binding capacity, or phosphorylation at Ser133 of CREB1 (upper middle). Hsa_circ_0026782 turns high‐transactivated CREB1 into low‐transactivated CREB1, and decreases the transcription of downstream target genes (upper right). By this mechanism, hsa_circ_0026782 inhibits keloids both in vitro and in vivo (lower).

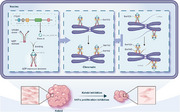

## 1 Introduction

Skin scarring is the final stage of wound healing in adults, characterized by the formation of a nonfunctional fibrotic tissue mass.^[^ ^1^, ^2^, ^3^, ^4^ ^]^ When abnormal wound healing occurs, it often leads to pathological scarring. Keloids are a common and representative form of pathological scars that can cause a series of complications, and are usually triggered by skin injuries or irritations that penetrate the reticular dermis. These injuries may stem from various sources, including trauma, burns, surgeries, vaccinations, acne, and so on.^[^ ^5^, ^6^, ^7^ ^]^ Clinicians define keloids as scars that spread beyond the original wound boundaries into the surrounding healthy skin, exhibiting tumor‐like invasiveness without resting or spontaneous regression phases.^[^ ^8^, ^9^, ^10^ ^]^ Histologically, keloids present a marked imbalance between the anabolic and catabolic phases, such as an increase in the number of inflammatory cells and fibroblasts in the reticular layer, enhanced angiogenesis, and excessive deposition of large bundles of disorganized arranged type I collagen fibers.^[^ ^8^, ^10^, ^11^ ^]^

There are no precise global statistics on the incidence rate of pathological scars yet. It is estimated that the incidence rate of scarring after surgery or burns can reach 30–70%,^[^ ^12^ ^]^ of which approximately 15% are pathological scars.^[^ ^13^ ^]^ Clinical interventions for keloids include traditional surgical treatments and nonsurgical approaches (such as laser therapy and topical corticosteroid injection).^[^ ^11^, ^14^, ^15^ ^]^ However, the recurrence rates for keloid excision surgery can reach 45–100%. Epidemiological studies suggest that genetic susceptibility plays a key role in keloid formation, as patients often report a positive family history.^[^ ^10^, ^16^ ^]^ Furthermore, keloids display a racial bias, with dark‐skinned individuals showing greater susceptibility.^[^ ^11^, ^16^ ^]^

The cyclic adenosine monophosphate (cAMP) response element‐binding protein (CREB1/CREB) is a kinase‐modulated transcription factor, and belongs to the basic leucine zipper (bZIP) family.^[^ ^17^ ^]^ CREB1 can form either homodimers or heterodimers with other bZIP family members via its bZIP domain.^[^ ^18^, ^19^ ^]^ Additionally, the bZIP domain is the DNA binding region.^[^ ^17^ ^]^ The cAMP response element (CRE) is a palindromic sequence consisting of eight nucleotides (5′‐TGACGT(C/A) (G/A)‐3′), found in the regulatory regions of CREB1 target genes.^[^ ^20^ ^]^ The key region associated with the transactivation of CREB1 is the kinase‐inducible domain (KID), which contains several serine residues (Ser) that serve as substrates for upstream kinases. When these serine residues are phosphorylated, the KID stably interacts with the coactivators CBP and/or p300, initiating the assembly of the transcriptional machinery.^[^ ^17^ ^]^

After being synthesized in the cytoplasm, CREB1 forms an inactive dimer and is subsequently transported to the nucleus.^[^ ^18^ ^]^ CREB1 can be activated by cAMP signaling or other stimuli through phosphorylation at specific Ser residues within the KID domain. Ser133 and Ser142 are the two major phosphorylation sites. Phosphorylation at Ser133 (p‐Ser133) enhances the transactivation of CREB1, leading to dynamic transcriptional regulation of numerous genes, which in turn endows cells with oncogenic properties.^[^ ^21^, ^22^, ^23^, ^24^ ^]^ In response to stimuli, some kinases, such as MAP kinase/ERK1/2 cascade and calcium/calmodulin‐dependent protein kinases (CaMKs), can mediate CREB1 phosphorylation at Ser133.^[^ ^25^ ^]^ In contrast, CREB1 phosphorylation at Ser142 (p‐Ser142) leads to decreased transactivation.^[^ ^26^ ^]^ However, the role of CREB1 phosphorylation at Ser142 and the mechanism behind its role remain elusive. Some studies have shown that p‐Ser142 is an additional posttranslational modification that occurs after p‐Ser133^[^ ^27^ ^]^ through an activity‐dependent mechanism.^[^ ^18^ ^]^

CircRNAs were initially regarded as “junk” produced by aberrant splicing events. However, later studies have demonstrated that circRNAs can exert diverse biological functions by acting as transcriptional regulators, microRNA (miRNA) sponges, and even protein templates.^[^ ^28^, ^29^, ^30^, ^31^, ^32^ ^]^ With recent advances in sequencing technologies and bioinformatics tools, studies on the roles of circRNAs in clinical diseases are rapidly expanding,^[^ ^33^, ^34^ ^]^ particularly in the field of wound healing and scarring. Recent studies have demonstrated that circRNAs can regulate wound repair and scar development through distinct molecular mechanisms. For instance, Chen et al. reported that circ‐ITCH recruits the RNA‐binding protein TAF15 to activate the Nrf2 signaling pathway, thereby inhibiting ferroptosis and promoting angiogenesis, ultimately accelerating the healing of diabetic foot ulcers.^[^ ^35^ ^]^ Oppositely, circ_PRKDC is shown to act as a competing endogenous RNA (ceRNA) for miR‐20a‐3p^[^ ^36^ ^]^ and miR‐31,^[^ ^37^ ^]^ modulating the proliferation and migration of keratinocytes and delaying wound closure in diabetic ulcers. In the context of scars (mainly in pathological, such as hypertrophic scars and keloids), circRNAs are predominantly reported to function as miRNA sponges, blocking miRNA‐mediated gene silencing and thus influencing fibrotic responses. A representative example is circCOL5A1, which acts as a sponge for miR‐877‐5p to regulate keloid progression via the circCOL5A1/miR‐877‐5p/EGR1 axis;^[^ ^38^ ^]^ or for miR‐7‐5p, thereby modulating the keloids through the circCOL5A1/miR‐7‐5p/Epac1 axis.^[^ ^39^ ^]^ These findings highlight the essential role of circRNAs in the regulation of wound repair, scars, and keloids through complex networks.

Up to now, the RNA sequencing of keloids mostly use normal skin tissues as a negative control for further analysis.^[^ ^40^, ^41^ ^]^ In this study, we used normal scars as a control group for high‐throughput sequencing to explore the potential mechanisms of the genetic heterogeneity of keloids. A novel circRNA, hsa_circ_00 26782, was found to be downregulated in keloids. Surprisingly, this circRNA did not play its conventional roles like miRNA sponge, or ceRNA, or encoding oligopeptides,^[^ ^39^, ^42^, ^43^, ^44^ ^]^ but directly bound to CREB1. Interestingly, hsa_circ_00 26782 increased the exposure of CREB1 bZIP domain to promote CREB1 dimerization; it also improved the phosphorylation at Ser142 of CREB1 to inhibit its interaction with promoters or enhancers of downstream genes. This led to a temporal inhibition of transactivated CREB1 to “switch off” the CREB1‐mediated transcription, and ultimately suppressed the progression of keloids both in vitro and in vivo, which indicates hsa_circ_00 26782 acts as a “molecular break” in keloid progression. Our study expands the understanding of the underlying pathological mechanisms of keloids, offering potential contributions to improving patient outcomes and quality of life.

## 2 Results

### 2.1 Compared with Normal Scar, Hsa_circ_00 60927 and Hsa_circ_0 003563 Are Upregulated in Keloids, While Hsa_circ_00 26782 and Hsa_circ_0000085 Are Downregulated

**Figure 1:**
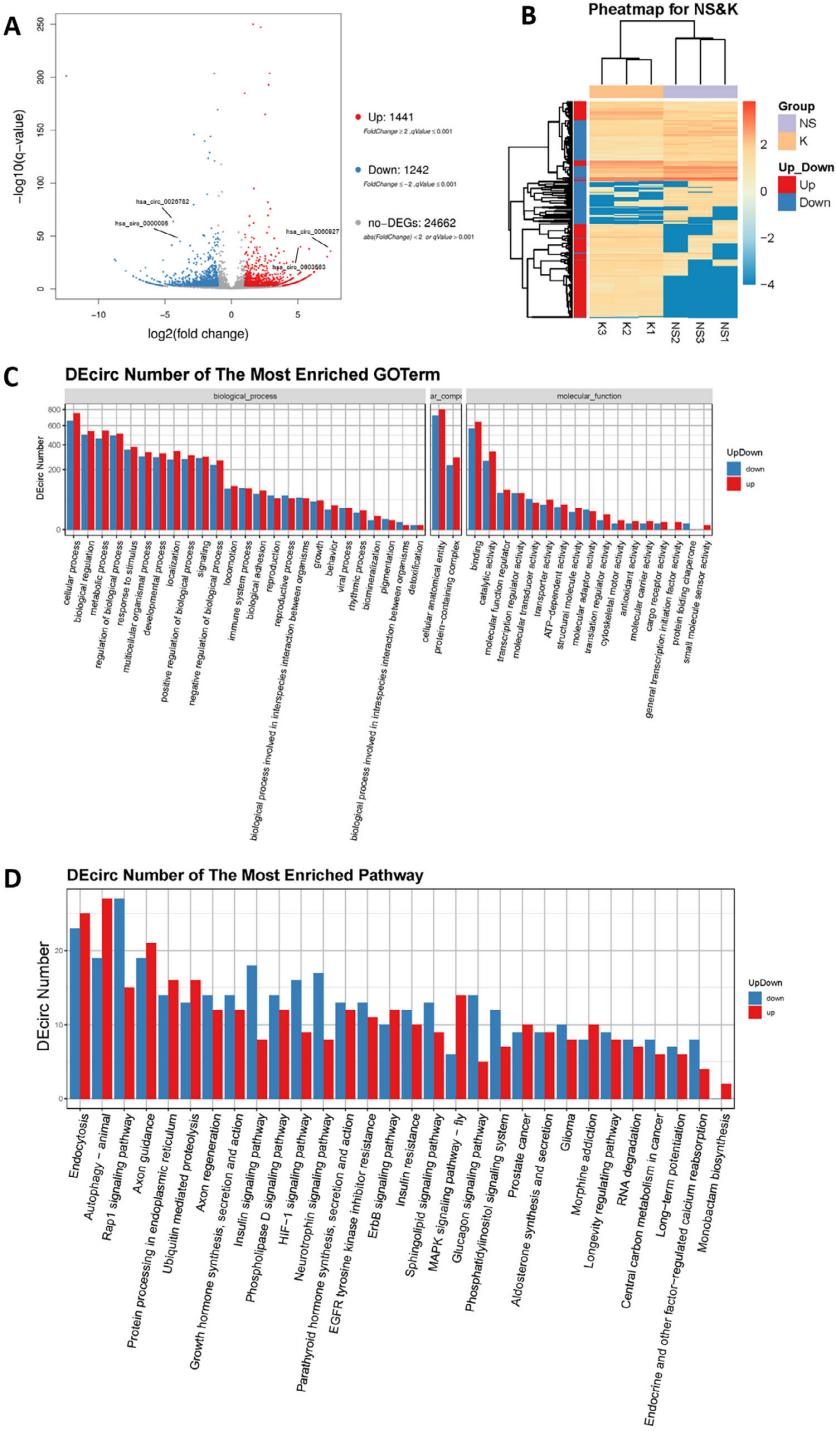
Differential analysis and enrichment analysis of DEcircs. (A) Volcano plot of DEcircs. Each plot represented a circRNA. Upregulated circRNAs in keloid were marked in red, downregulated circRNAs were marked in blue, and the gray plot represented non‐differentially expressed circRNAs. (B) Pheatmap of DEcircs. The X‐axis represented samples from the cluster analysis, and the Y‐axis represented DEcircs. Colors represent log10‐transformed expression (darker colors indicated higher expression, and lighter colors indicated lower expression). (C) GO enrichment analysis of DEcircs. The X‐axis represented the GO functional classification, and the Y‐axis represented the number of upregulated or downregulated circRNAs in the corresponding GO term. (D) KEGG enrichment analysis of DEcircs. The X‐axis represented the pathway entry, and the Y‐axis represented the number of upregulated or downregulated circRNAs in the corresponding pathway entry.

To characterize the circRNA expression profiles and explore their roles in genetic heterogeneity of keloids, we compared three pairs of keloids (excised from keloid patients) and normal scars (excised from patients who received removal of internal fixation for fractures) using circRNA high‐throughput sequencing. A total of 17,566 circRNAs were identified (Figure S1A, Supporting Information), which widely distributed throughout the whole genome (Figure S1B, Supporting Information). Among these circRNAs, 1,441 circRNAs were significantly upregulated in keloid tissues, whereas 1,242 circRNAs were downregulated (*q*‐value < 0.001 and fold change> 2; **Figure** **1**A). A heatmap was generated to illustrate the expression levels of circRNAs in each tissue (Figure 1B).

On basis of the sequencing analysis, we performed Gene Ontology (GO) enrichment analysis on the differentially expressed circRNAs (DEcircs). The GO enrichment analysis categorized the DEcircs into three main functional classes: molecular function, cellular component, and biological process. Notably, the most abundant DEcircs were associated with the cellular process of biological process (1416 DEcircs), cellular anatomical entity of cellular component (1526 DEcircs), and binding of molecular function (1209 DEcircs) (Figure 1C). KEGG enrichment analysis further revealed that the DEcircs were most prominently enriched in the autophagy and endocytosis pathways (Figure 1D).

**Figure 2:**
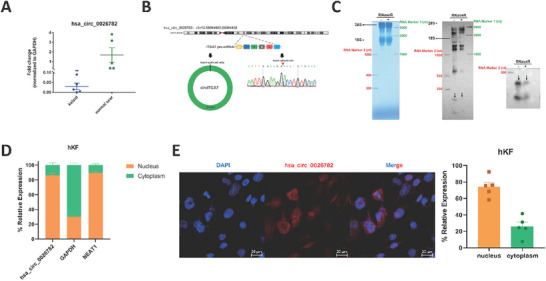
Hsa_circ_00 26782 is upregulated in hKFs and mainly locates in the nucleus. (A) The expression levels of hsa_circ_00 26782 in five pairs of keloids (blue) and normal scars (green). (B) Genome location of the coding gene of hsa_circ_00 26782, components of hsa_circ_00 26782, and Sanger sequencing of the RT‐qPCR product of hsa_circ_00 26782. The BSJ site was marked by a red inverted triangle. (C) Northern blot was performed on RNase R‐digested or undigested total RNA samples. The hsa_circ_00 26782 bands were indicated by black arrows. (D) Expression within hKFs was detected by RT‐qPCR. (E) Subcellular location of hsa_circ_00 26782 was verified by RNA FISH through specific probe (red). Cell nuclei were stained with DAPI (blue). Fluorescence image (left) and quantification of subcellular components (right). Original magnification: 200×. All experiments were performed in triplicate. The error bars indicate the mean ± SD. ns *p* > 0.05, **p < 0.01, ***p < 0.001, ****p < 0.0001 by independent t‐tests.

Many novel circRNAs unverified yet were predicted in this analysis. There is a high probability that these circRNAs are derived from other transcripts. Therefore, we focused our subsequent research on DEcircs already recorded in CircBase (labeled with the prefix “hsa”). By considering both the expression differences (log2 ratio) and relative expression levels, we selected the top ten upregulated DEcircs (hsa_circ_00 60927, hsa_circ_0 003563, hsa_circ_00 71408, hsa_circ_0 002132, hsa_circ_0 008013, hsa_circ_0 004874, hsa_circ_0 007279, hsa_circ_00 30034, hsa_circ_0 004606, and hsa_circ_00 73244), and three downregulated DEcircs (hsa_circ_00 26782, hsa_circ_0000095, and hsa_circ_0 005757) in keloids, to verify their expression levels in six pairs of keloids and normal scars. RT‐qPCR showed that the expression differences of these circRNAs between keloids and normal scars also existed in other patients, suggesting this might be a common phenomenon for keloids (**Figures** **2**A, S2A, Supporting Information). From these thirteen circRNAs, we chose four DEcircs with more significant differences in expression level between keloids and normal scars (hsa_circ_00 60927, hsa_circ_0 003563, hsa_circ_00 26782, and hsa_circ_0000095) for further genomic alignment analysis. According to the genomic alignment in UCSC Genome Browser, these four circRNAs are exons derived from different transcripts (ecircRNAs). Specifically, hsa_circ_00 60927 consists of exons 3–11 of the *CYP24A1* gene, which is located on chromosome 20q13.2 (Figure S2B, Supporting Information); hsa_circ_0 003563 is formed by exons 3 and 4 of the *RUNX2* gene, located on chromosome 6p21.1 (Figure S2B, Supporting Information); hsa_circ_00 26782 originates from the head‐to‐tail junction of exon 4 of the *ITGA7* gene and is located on chromosome 12q13.2 (Figure 2B); hsa_circ_0000095 is composed of exons 2–5 of the *TMEM56‐RWDD3* gene, located on chromosome 1p21.3 (Figure S2B, Supporting Information).

In contrast to linear RNAs, one of the typical characteristics of circRNAs is their resistance to RNase R digestion. We treated total RNA samples with RNase R and performed RT‐qPCR to validate the circRNAs. The PCR products were then analyzed via Sanger sequencing and agarose gel electrophoresis. Sanger sequencing confirmed the back‐splicing junction (BSJ) sites, which were consistent with the BSJ sequences of the DEcircs in CircBase (Figure 2B, Figure S2B, Supporting Information). The agarose gel results showed that the four selected DEcircs, rather than GAPDH linear RNA, could resist RNase R digestion (Figure S3 left, Supporting Information). Since circRNAs are generated from pre‐mRNAs through back‐splicing, another typical characteristic of circRNAs is that the BSJ sequences do not exist in the real genome. Therefore, we conducted RT‐qPCR experiments on cDNA and genomic DNA (gDNA) using divergent or convergent primers, followed by agarose gel electrophoresis of the reaction products. The electrophoresis results showed that the BSJ sequences of these four DEcircs were absent in the gDNA (Figure S3 right, Supporting Information), whereas corresponding circRNAs with high specificity appeared in the amplification with the divergent primers, and nonspecific bands of similar transcripts appeared in the convergent primers. The results further confirmed the identity of these circRNAs.

### 2.2 Hsa_circ_00 26782 Is Mainly Located in the Nucleus of hKFs

To investigate the roles of these DEcirs in the keloids, we explored the subcellular location of hsa_circ_00 26782. The hKFs were sampled from human keloids (Figure S4A, Supporting Information), and the cytoplasmic components and nucleic components were then separated. The RT‐qPCR showed that average 86.25% of hsa_circ_00 26782 was from the nucleus, while average 13.85% was from the cytoplasm, indicated that hsa_circ_00 26782 was predominantly located in the nucleus (Figure 2D). A specific probe was designed to exam the hsa_circ_00 26782, but not the linear transcript of *ITGA7* exon4 in CircBase. RNA fluorescence in situ hybridization (FISH) assay with this specific probe also displayed that although hsa_circ_00 26782 was distributed in both the cytoplasm and the nucleus, it was mainly located in the nucleus of most hKFs, and the fluorescence intensity in the nucleus was significantly higher than that in cytoplasm. Specifically, about 74.24% of endogenous hsa_circ_00 26782 signals were localized in the nucleus (Figure 2E), confirming the results of RT‐qPCR.

### 2.3 Hsa_circ_00 26782 Inhibits Cell Proliferation, Migration and Invasion of hKFs, Promotes Cell Apoptosis, and Stocks Cell Cycle in S Phase

**Figure 3:**
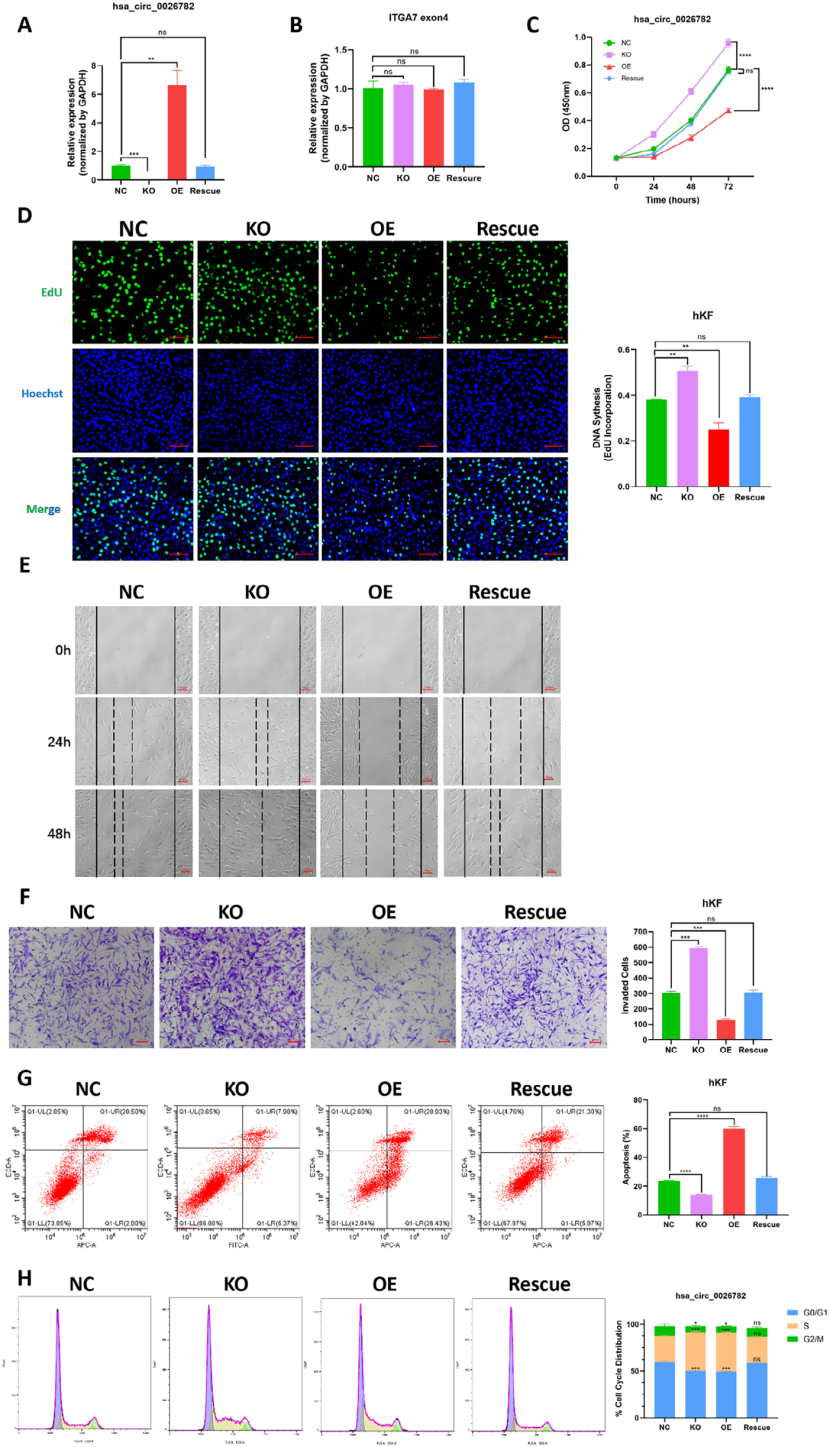
Hsa_circ_00 26782 inhibits cell proliferation, migration, and invasion, promotes cell apoptosis, stocks cell cycle in S phase of hKFs. (A) Knockout and overexpression efficiency of hsa_circ_00 26782 in hKFs. “NC” represented untreated hKFs; “KO” represented hKFs in which hsa_circ_00 26782 expression was knocked out. “OE” represented hKFs that stably overexpress hsa_circ_00 26782. “Rescue” represented HKFs in which the endogenous expression of hsa_circ_00 26782 was knocked out and supplemented with exogenous expression stably. (B) Expression level of ITGA7 exon 4 in the indicated hKFs. (C) CCK‐8 proliferation assay was performed on the indicated hKFs. The absorbance at OD450 was detected at 24, 48 and 72 hours after cell seeding. (D) EdU proliferation assay of the indicated hKFs. Fluorescence image (left) and quantification (right) of EdU‐positive hKFs. Cell nuclei were stained with Hoechst (blue). Original magnification: 200 × (E) Wound scratch assay of the indicated hKFs. The wound closure area was photographed at 0, 24, and 48 hours after cell seeding. (F) Transwell invasion assay of the indicated hKFs (left) and quantification of invasive cells (right). (G) Flow cytometry analysis of apoptosis in the indicated hKFs (left) and quantification of the percentage of apoptotic cells (right). (H) Flow cytometry analysis of cell cycle distribution of the indicated hKFs (left) and quantification of the cell cycle distribution (right). All experiments were performed in triplicate. The error bars indicate the mean ± SD. ns *p* > 0.05, **p < 0.01, ***p < 0.001, ****p < 0.0001 by independent t‐tests.

Since hsa_circ_00 26782 was downregulated in keloids compared with normal scars, we were wondering if hsa_circ_00 26782 plays a role in keloid formation. Three nearest Alu elements were located upstream and downstream of hsa_circ_00 26782, including one upstream Alu element (AluJo) and two downstream Alu elements (AluJb and AluSq2). Using CRISPR/Cas9 technique, we mutated two of three Alu elements or all three Alu elements in hKFs. The knockdown efficiency of hsa_circ_00 26782 was assessed by RT‐qPCR. We found that knockout of both AluJo and AluSq2 elements reached the maximum knockdown efficiency of hsa_circ_00 26782 (Figure S4B, Supporting Information). Therefore, sgRNA targeting both AluJo and AluSq2 was applied in subsequent experiments. Considering the unexpected off‐target effects caused by long‐term expression of the Cas9 protein, we transiently transfected the CRISPR/Cas9 plasmid in hKFs and then performed fluorescence activated cell sorting (FACS) to generate the hKFs with hsa_circ_00 26782 knockout (KO). The overexpression plasmid, pLCDH‐CiR, carrying linear sequence of hsa_circ_00 26782, was used to construct the hKFs with stably over‐expressed hsa_circ_00 26782 (OE) by lentivirus and sorted by FACS. The linear sequence of hsa_circ_00 26782 would be cyclized by the circular fragments within the plasmids. The overexpression plasmid pLCDH‐CiR was also transfected into the hKFs with hsa_circ_00 26782 knockout (KO) by lentivirus for the rescue experiments. RT‐qPCR showed that compared with NC group, the expression of hsa_circ_00 26782 in OE group was increased by 6.63 times, but it was almost undetectable in KO group (**Figure** **3**A).

We firstly investigated whether hsa_circ_00 26782 influenced the expression of its host gene *ITGA7* exon 4. The RT‐qPCR results indicated that the back‐splicing process of hsa_circ_00 26782 did not affect the production of linear *ITGA7* exon 4 (Figure 3B). CCK‐8 proliferation assay, immunofluorescence staining of Ki67, and EdU assay were then performed to investigate the role of hsa_circ_00 26782 in cell proliferation. The results showed that depletion of hsa_circ_00 26782 significantly promoted the proliferation frequency of hKFs in vitro, while overexpression of hsa_circ_00 26782 had the opposite effect (Figure 3C,D, Figure S4D,E, Supporting Information). To investigate the role of hsa_circ_00 26782 in cell migration and invasion, wound healing assay and transwell invasion assay were performed. The data showed that hsa_circ_00 26782 exerted an inhibitory effect on migration (Figures 3E and S4F, Supporting Information) and invasion ability of hKFs (Figures 3F and S4G, Supporting Information).

Given the important role of apoptosis in the keloid formation, we investigated whether hsa_circ_00 26782 could influence apoptosis in hKFs. Flow cytometry analysis showed that the percentage of apoptotic cells in hKFs was increased by hsa_circ_00 26782 overexpression, and significantly depressed by hsa_circ_00 26782 depletion (Figure 3G, Figure S4H, Supporting Information).

The cell cycle is an important indicator of cell proliferation and tumorigenicity, so we performed flow cytometry to analyze the effect of hsa_circ_00 26782 on cell cycle. Unexpectedly, the analysis displayed that percentage of cells in the S phase was significantly increased in both the KO group (49.9% ± 0.9%) and the OE group (49.25% ± 0.65%), compared with that in the NC group (28% ± 0.9%) and the Rescue group (27.65% ± 0.95%) (Figure 3H), which was inconsistent with the results of the cell proliferation analyses (the KO‐hKFs presented a significant increase in proliferation, whereas the OE‐hKFs presented significant inhibition of proliferation). One of possible reasons was that the increase of the percentage of S phase cells in the KO‐hKFs was caused by active cell proliferation, while the increase of the percentage of S phase cells in the OE‐hKFs was due to S phase arrest.

### 2.4 Compared with Hsa_circ_00 26782, Hsa_circ_00 60927 and Hsa_circ_0 003563 Exhibit Opposite Biological Functions, While Hsa_circ_0000095 Exhibits Similar Biological Functions

We simultaneously examined the biological functions of hsa_circ_00 60927, hsa_circ_0 003563, and hsa_circ_0000095 on hKFs. Two circRNAs upregulated in keloids, hsa_circ_00 60927 and hsa_circ_0 003563, were knocked down and overexpressed by corresponding siRNAs or pLCDH‐CiR plasmids, while hsa_circ_0000095 downregulated in keloids was only overexpressed due to its low expression level in hKFs (Figure S4C, Supporting Information). CCK‐8 assay and Ki67 immunofluorescence staining showed that hsa_circ_00 60927 and hsa_circ_0 003563 enhanced the proliferation, migration, and invasion abilities of hKFs and inhibited apoptosis (Figure S4D–H, Supporting Information). In contrast, hsa_circ_0000095 exhibited biological functions similar to those of hsa_circ_00 26782 (Figure S4D–H, Supporting Information).

The results indicate that upregulated circRNAs in keloids promote keloid formation, while downregulated circRNAs in keloids inhibit keloid formation.

### 2.5 Hsa_circ_**00** **26782** Inhibited Keloid Progression in Vivo

After comparing the phenotypic effects of the four DEcircs on hKFs, we found that hsa_circ_00 26782 exhibited comparable or even more pronounced biological effects than the other three DEcircs, even at the lowest level of overexpression (Figure S4C, Supporting Information). In addition, compared to hsa_circ_0000095, we also noted that hsa_circ_00 26782, although significantly downregulated in keloids, could not be efficiently overexpressed using plasmid‐based methods through transient transfection or lentiviral infection, which drew our attention. Therefore, we selected hsa_circ_00 26782 for subsequent investigation into its molecular mechanisms.

**Figure 4:**
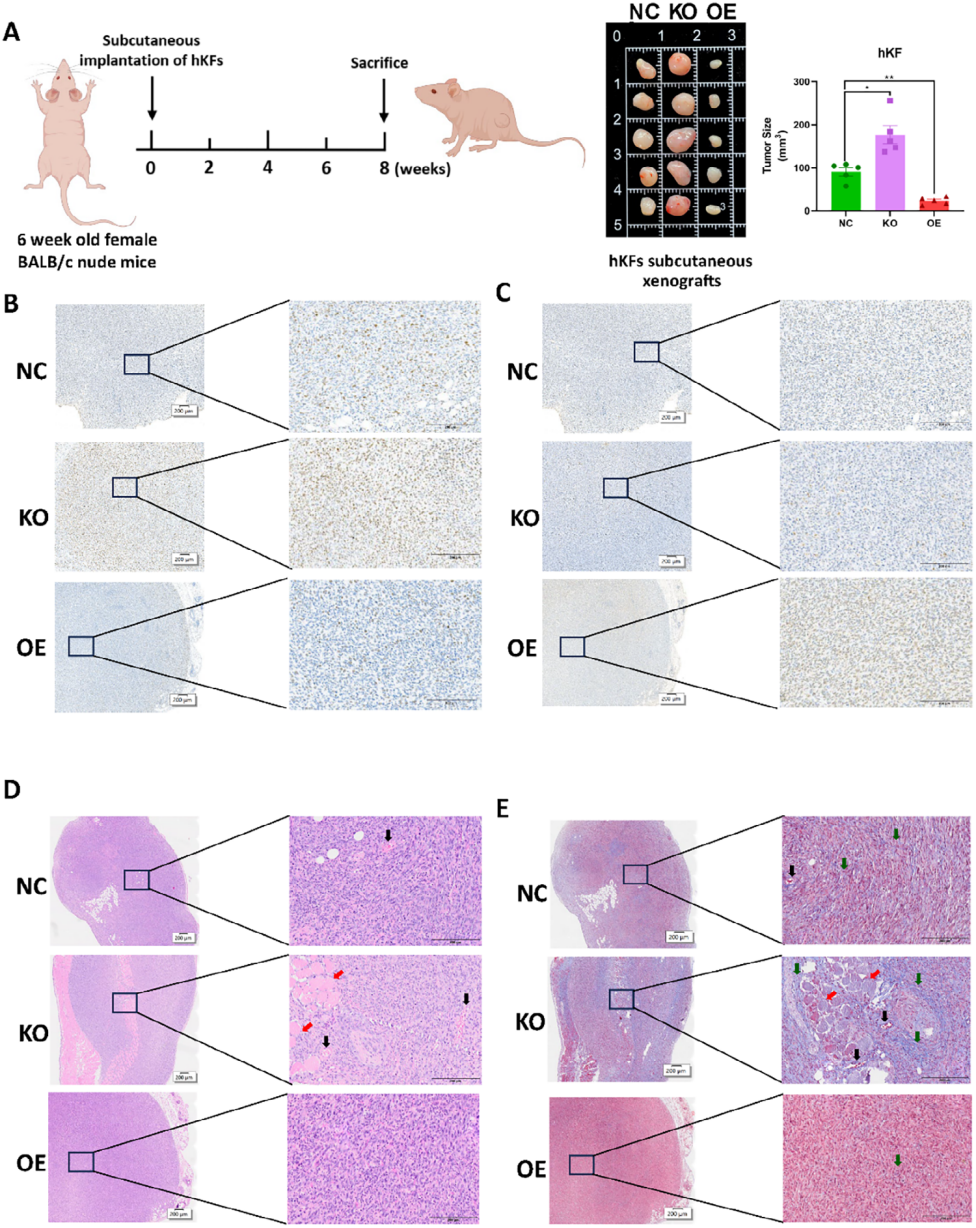
Hsa_circ_00 26782 inhibited keloids in vivo. (A) Six‐week‐old female BALB/c nude mice were subcutaneously injected with 1 × 10^7^ indicated hKFs (left). Animals were sacrificed at week eight, and the xenografts were isolated (middle) and measured (right). (B) Ki67 immunohistochemical staining of xenografts excised from nude mice. Scale bar, 200 µm. (C) TUNEL assay of xenografts excised from nude mice. Scale bar, 200 µm. (D) H&E staining of xenografts excised from nude mice. The red arrows indicated muscle fibers, and the black arrows indicated nascent small blood vessels. Scale bar, 200 µm. (E) Masson's staining of xenografts excised from nude mice. The red arrows indicated muscle fibers, the black arrows indicated nascent small blood vessels, and the green arrows indicated collagen fiber deposition. Scale bar, 200 µm. Three independent experiments with three technical repetitions were performed. The error bars indicate the mean ± SD. ns *p* > 0.05, *p < 0.05, **p < 0.01, ***p < 0.001 by independent t‐tests.

To verify whether hsa_circ_00 26782 inhibits keloid progression in vivo, we subcutaneously injected equal amounts of hKFs with different expression levels of hsa_circ_00 26782 into five female BALB/c nude mice (Figure S5, Supporting Information). The xenograft volumes were then measured and calculated. Compared with the NC group, the KO group presented a significant increase in tumor volume, while the OE group exhibited a dramatic reduction (**Figure** **4**A). In addition, we further validated the in vivo effects of hsa_circ_00 26782 on cell proliferation and apoptosis using Ki67 immunohistochemical staining and TUNEL assay in xenograft tumors. The results showed that overexpression of hsa_circ_00 26782 significantly inhibited cell proliferation (Figure 4B) and promoted apoptosis (Figure 4C) in the xenograft tumors, whereas knockout of hsa_circ_00 26782 produced the opposite effects. These results suggested that hsa_circ_00 26782 inhibited keloid growth in vivo.

The typical characteristic of keloids is the excessive deposition of collagen. Thus, we performed H&E staining and Masson's staining on xenograft tumors excised from nude mice. The H&E staining results showed that, compared with the NC group, the formation of new small vessels and myofibers within the tumors increased in the KO group, while almost no small vessels and myofibers were observed in the OE group (Figure 4D). The Masson's staining results also demonstrated that the KO group presented substantial deposition of gray‐green stained collagen accompanied by the formation of a considerable amount of pink‐stained myofibers. In contrast, the OE group showed only minimal collagen deposition with almost no myofiber formation (Figure 4E). These staining results suggested that hsa_circ_00 26782 might inhibit the pathological progression of keloids in vivo.

### 2.6 Hsa_circ_**00** **26782** Binds to CREB1 through 1–90 Nucleotides (nt)

Next, we investigated the molecular mechanism behind of the function of hsa_circ_00 26782. We first validated the authenticity of hsa_circ_00 26782 using the gold standard for circRNA detection—the northern blot assay. The hybridization with the BSJ‐specific biotin‐labeled probe showed that hsa_circ_00 26782 was resistant to RNase R digestion, with a size of approximately 256 nt, consistent with the predicted size in the CircBase and circPrimer databases (Figure 2C).

**Figure 5:**
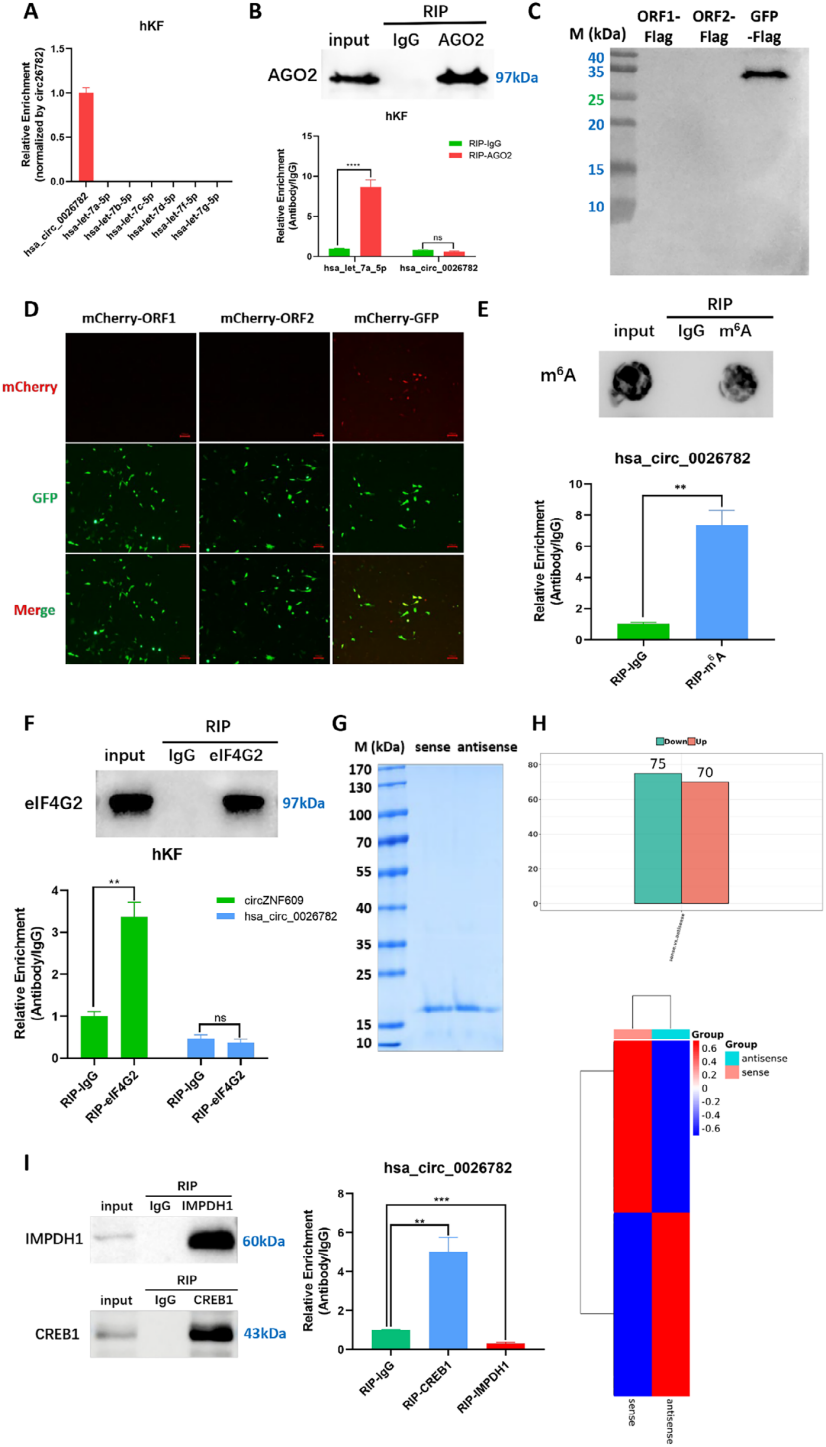
Hsa_circ_00 26782 binds to CREB1 through 1–90 nucleotides (nt). (A) Prediction of miRNA that might bind to hsa_circ_00 26782 by using miRanda and RNAhybrid. Hsa‐let‐7a‐5p may potentially bind to hsa_circ_00 26782 but with a *p*‐value> 0.05. (B) ORF prediction diagram of hsa_circ_00 26782 (upper) and details of the predicted ORFs and IRES (lower). (C) Western blot of the indicated flag‐tagged oligopeptides purified via an IP assay. (D) Fluorescence signals of mCherry and GFP were observed under a fluorescence microscope. (E) Dot blot of RIP assay using anti‐m^6^A antibodie (upper), and RT‐qPCR analysis of hsa_circ_00 26782 pulled down by anti‐m^6^A antibodie (lower). (F) SDS‐PAGE of RIP assay using anti‐eIF4G2 antibodie (upper), and RT‐qPCR analysis of hsa_circ_00 26782 pulled down by anti‐eIF4G2 antibodie (lower). CircZNF609 was used as positive control. (G) Coomassie brilliant blue staining of the SDS‐PAGE gel of the proteins pulled down by RNA pulldown assay. (H) Differential analysis (upper) and heatmap (lower) of proteins pulled down by RNA pulldown assay. (I) SDS‐PAGE of RIP assay using the indicated antibodies (left), and RT‐qPCR analysis of hsa_circ_00 26782 pulled down by indicated antibodies (right). All experiments were performed in triplicate. Error bars indicate the mean ± SD. ns *p* ＞ 0.05, **p < 0.01, ***p < 0.001 by independent t‐tests.

Given that the most well‐characterized function of circRNAs is their role as miRNA sponges or ceRNAs, we used miRanda and RNAhybrid software to predict potential miRNA interactions with DEcircs. A total of 2562 miRNAs were identified as having direct interactions with DEcircs. The interaction network was visualized via R language (Figure S6, Supporting Information). To investigate whether hsa_circ_00 26782 might play a role in this process, we merged the results of DEcirc‐miRNA interaction network, miRanda database, and RNAhybrid database. The prediction results indicated that only one miRNA, hsa‐let‐7a‐5p, could potentially bind to hsa_circ_00 26782, but with no statistical significance (*p*‐value> 0.05; Figure S7A, Supporting Information). Due to the high sequence homology among members of the hsa‐let‐7 family (such as let‐7a‐5p, let‐7b‐5p, let‐7c‐5p, let‐7d‐5p, let‐7f‐5p, and let‐7g‐5p), we performed an RNA‐RNA pulldown assay using biotin‐labeled hsa_circ_00 26782 as probes in hKFs, and analyzed the RNAs bound to the probe through RT‐qPCR. The results showed that hsa‐let‐7 family members mentioned above were not enriched, indicating that hsa_circ_00 26782 and hsa‐let‐7 family members may not directly interact with each other (**Figure** **5**A).

Then, we utilized the circInteractome,^[^ ^45^ ^]^ RBPsuite,^[^ ^46^ ^]^ and CRWS^[^ ^47^ ^]^ databases to assess whether hsa_circ_00 26782 interact with Argonaute 2 (AGO2), the major component of the RNA‐induced silencing complex (RISC) in RNA interference (RNAi) of miRNAs.^[^ ^48^, ^49^ ^]^ The circInteractome database predicted no direct interactions between hsa_circ_00 26782 and AGO2. CRWS database predicted 27 potential binding sites of AGO2 on hsa_circ_00 26782 (score> 0.8; Figure S7B, Supporting Information). However, the lengths of the predicted binding sites in CRWS were concentrated around 1 nt, and such a short length can hardly mediate a stable binding interaction. And the RBPsuite showed that although three potential binding sites between hsa_circ_00 26782 and AGO2 were predicted, only one binding site with a score greater than 0.5 (score = 0.57094866), suggesting a low likelihood of interaction (Figure S7C, Supporting Information). For the reason of the predictions in different databases were paradoxical, we performed RNA immunoprecipitation (RIP) by using proteinA/G magnetic beads coated with antibody against AGO2 or corresponding IgG (negative control) to pulldown RNAs bound to AGO2, and validated specific RNAs binding to AGO2 through qRT‐PCR assay. The result showed that hsa‐let‐7a‐5p (positive control) was found bound to AGO2, but hsa_circ_00 26782 was not detected in RT‐qPCR (Figure 5B). This result also indicated that hsa_circ_00 26782 did not bind to hsa‐let‐7a‐5p. Based on the above results, we excluded the possibility that hsa_circ_00 26782 functions as a miRNA sponge or ceRNA.

Another emerging mechanism is that some circRNAs are capable of translating oligopeptides or proteins via IRES and ORF‐dependent mechanism or m^6^A modification‐mediated mechanism. To explore this potential in hsa_circ_00 26782, we used circPrimer, CIRCpedia, and CircBase to predict possible IRES sites and ORFs. The integrated prediction results revealed two potential ORFs in hsa_circ_00 26782, which encode peptides of 71 amino acids and 141 amino acids in length, respectively (Figure S7D, Supporting Information). To validate the conjecture, we inserted a Flag‐tag sequence (GATTACAAGGACGACGATGACAAG) immediately after the start codon (AUG) of the two ORFs of hsa_circ_00 26782 in the overexpression vector, respectively. The Flag sequence was also inserted after the start codon of GFP in the empty pLCDH‐CiR vector as a positive control. Three recombinant plasmids were then transfected into hKFs, and immunoprecipitation (IP) was performed using Flag beads to enrich Flag‐tagged oligopeptides. On basis of the lengths of the ORFs, we estimated that the ORF1‐Flag fusion protein, ORF2‐Flag fusion protein, and GFP‐Flag fusion protein would be approximately 8.9, 16.5, and 27.9 kDa, respectively. Western blot analysis showed that the GFP‐Flag fusion protein was detectable via an anti‐Flag antibody, whereas the proteins corresponding to ORF1 and ORF2 were not detected (Figure 5C). Additionally, we inserted the mCherry sequence after the start codons in the same way as the Flag‐tag, enabling the vectors to express mCherry‐ORF or mCherry‐GFP fusion proteins. The fluorescence signals were observed under a fluorescence microscope after transient transfection. The results showed that GFP fluorescence was observed in all groups, indicating successful transfections. However, red mCherry fluorescence was observed only in the GFP‐mCherry group, while no fluorescence was detected in either the ORF1‐mCherry or ORF2‐mCherry group (Figure 5D). These results suggested that hsa_circ_00 26782 could not express oligopeptides.

Previous studies have demonstrated that a single m^6^A site is sufficient to initiate circRNA translation. Therefore, we used circPrimer to predict the possible m^6^A modification sites of hsa_circ_00 26782, and the prediction showed five potential modification sites (Figure S7E, Supporting Information). Methylated RNA immunoprecipitation (MeRIP) was performed by using proteinA/G magnetic beads coated with m^6^A specific antibodies or corresponding IgG (negative control) to verify whether hsa_circ_00 26782 could be modified by m^6^A. The RT‐qPCR result showed that hsa_circ_00 26782 could be modified by m^6^A, which indicated that hsa_circ_00 26782 might drive the translation of oligopeptides or proteins through this way (Figure 5E). Nevertheless, a translational profiling study of the human heart based on Ribo‐seq analysis confirmed that hsa_circ_00 26782 was not detected on ribosomes, suggesting that it might lack the capacity for peptide translation.^[^ ^50^ ^]^ The m^6^A‐driven translation process of circRNAs requires the involvement of eukaryotic translation initiation factor 4 gamma 2 (eIF4G2; one of the key components of ribosomes) and the m^6^A reader YTH domain family protein 3 (YTHDF3).^[^ ^51^ ^]^ To confirm whether hsa_circ_00 26782 undergoes m^6^A‐mediated translation in hKFs, we performed a RIP assay using eIF4G2‐specific antibody, followed by RT‐qPCR to assess the interaction between hsa_circ_00 26782 and eIF4G2. The RIP assay indicated that there was no significant difference between eIF4G2 and IgG on hsa_circ_00 26782 enrichment; whereas compared to the negative control IgG, eIF4G2 robustly enriched circZNF609 (positive control). CircZNF609 is a well‐characterized circRNA known to be translated into functional oligopeptides via m^6^A‐dependent mechanism.^[^ ^52^, ^53^, ^54^ ^]^ These results demonstrated that hsa_circ_00 26782 was not directly enriched by eIF4G2, suggesting that hsa_circ_00 26782 was unlikely to engage with ribosomes and undergo m^6^A‐mediated translation (Figure 5F).

After excluding the potential role of hsa_circ_00 26782 as a miRNA sponge or ceRNA or encoding oligopeptides, we turned our attention to explore whether hsa_circ_00 26782 exerted its biological function through interactions with RNA‐binding proteins (RBPs). To verify this hypothesis, we performed an RNA pulldown assay using biotin‐labeled hsa_circ_00 26782 as probes to isolate RBPs that bind to hsa_circ_00 26782 in hKFs. The pulled‐down proteins were applied to sodium dodecyl sulfate‐polyacrylamide gel electrophoresis (SDS‐PAGE; Figure 5G), and followed by mass spectrometry analysis on the retrieved proteins. The mass spectrometry results showed that 70 proteins were significantly enriched by the hsa_circ_00 26782 sense RNA, compared with the antisense RNA (acted as negative control; Figure 5H).

Subsequently, the RBPs enriched by the sense RNA were subjected to GO enrichment and KEGG pathway analyses. The GO enrichment analysis (Figure S7F, Supporting Information) showed that hsa_circ_00 26782 associated RBPs were enriched primarily in translation (85 RBPs), cytoplasmic translation (73 RBPs), and mRNA splicing (56 RBPs) of the biological process category; and mainly in the cytosol (542 RBPs), nucleus (479 RBPs), and extracellular exosome (453 RBPs) of cellular component category; and in the functions of RNA binding (376 RBPs), identical protein binding (172 RBPs), and ATP binding (157 RBPs) of the molecular function category. KEGG pathway enrichment analysis highlighted that the most significantly enriched pathways were related to the ribosome, COVID‐19, and spliceosome (Figure S7G, Supporting Information).

After ranking the differential binding multiples, we selected the top 10 proteins showing the greater fold changes among the 70 proteins with increased association to the sense RNA. The commonly encountered background proteins frequently observed in mass spectrometry analyses (such as keratin 83 and keratin 36) were excluded, and taking into consideration the biological functions of hsa_circ_00 26782, we focused on inosine‐5‐monophosphate dehydrogenase 1 (IMPDH1; top 1) and CREB1 (top 5) for further investigation. Given that a linearized RNA sequence of hsa_circ_00 26782 was used as the probe in the RNA pulldown assay, which may differ from the actual circular circRNA, we conducted a RIP assay to validate the interaction between hsa_circ_00 26782 and IMPDH1 or CREB1. In the RIP assay, protein A/G magnetic beads coated with antibodies against the proteins or corresponding IgG (negative control) were used to pull down the bound RNAs, and the presence of hsa_circ_00 26782 was detected using RT‐qPCR. The results showed that hsa_circ_00 26782 was successfully pulled down and enriched in the beads coated with the CREB1 antibody, but not in the beads coated with the IMPDH1 antibody or IgG (Figure 5I). These findings suggested that CREB1 directly interacted with hsa_circ_00 26782.

**Figure 6:**
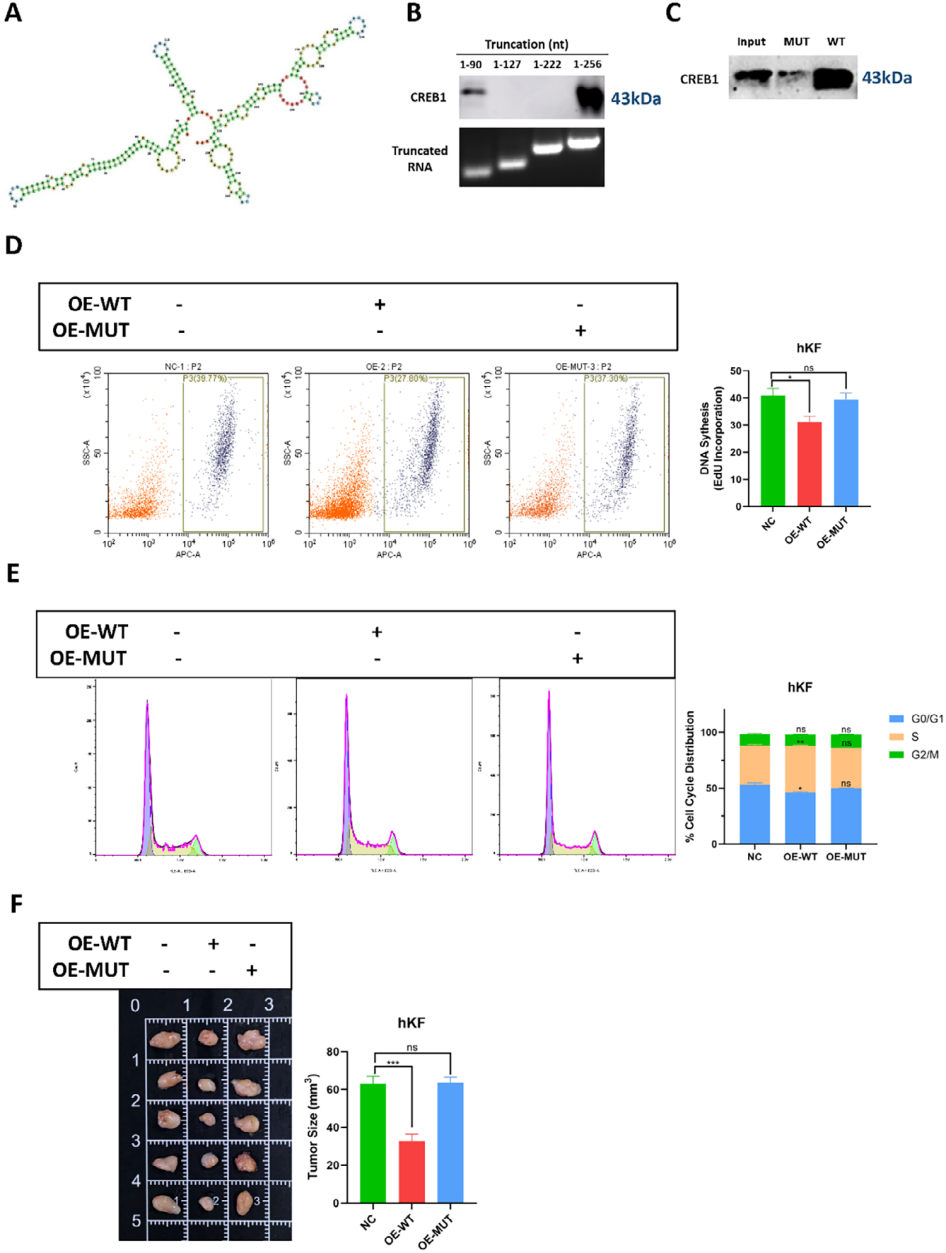
Hydrogen bonds also contribute to the hsa_circ_0026782‐CREB1 binding. (A) MFE plain secondary structure of complete hsa_circ_00 26782. (B) SDS‐PAGE of RNA pulldown assay of the indicated truncations (upper) for CREB1, and RNA agarose gel electrophoresis of the indicated truncations (lower). (C) SDS‐PAGE of CREB1 pulled down by mutant hsa_circ_00 26782 (MUT) or wild‐type hsa_circ_00 26782 (WT) in an RNA pulldown assay. (D) Flow cytometry analysis of EdU cell proliferation assay. “OE‐MUT” represented hKFs overexpressing mutant hsa_circ_00 26782, and “OE‐WT” represented hKFs overexpressing wild‐type hsa_circ_00 26782. The yellow dots represented empty hKFs, the purple dots represented EdU‐incorporated hKFs (left), and the quantification of EdU incorporation (right) was shown. (E) Flow cytometry analysis of cell cycle (left) and quantification of the percentage of cells in each cell cycle (right). (F) Six‐week‐old female BALB/c nude mice were subcutaneously injected with 1 × 10^7^ indicated hKFs. Animals were sacrificed at week eight, and the xenografts were isolated (left) and measured (right). Three independent experiments with three technical repetitions were performed. The error bars indicate the mean ± SD. ns *p* ＞ 0.05, *p < 0.05, **p < 0.01, ***p < 0.001 by independent t‐tests.

CircRNAs typically interact with RBPs through stem‐loop structures. To investigate the specific binding sites of hsa_circ_00 26782 with CREB1, we predicted the secondary structure of hsa_circ_00 26782 via RNAfold. Four major stem‐loops within hsa_circ_00 26782 were predicted, and typically distributed over 1–90, 90–127, 127–222, and 222–256 nt (**Figure** **6**A). We constructed truncations of hsa_circ_00 26782 containing different stem‐loops, and used these truncations as RNA pulldown probes. The results showed that only the truncation containing 1–90 nucleotides, or the full‐length sequence of hsa_circ_00 26782, could effectively pull down CREB1. Unexpectedly, despite containing the sequence of 1–90 nt, the truncations containing 1–127 or 1–222 nt were unable to pull down CREB1 (Figure 6B). To further confirm the interaction, we constructed a mutant hsa_circ_00 26782 by substituting adenine (A) with guanine (G), and thymine (T) with cytosine (C) within the 1–90 nt RNA sequences. RNA pulldown assay showed that, unlike wild‐type (WT) hsa_circ_00 26782, the mutant (MUT) hsa_circ_00 26782 could hardly pulled down CREB1 (Figure 6C).

### 2.7 The Interaction Between Hsa_circ_00 26782 and CREB1 Is Required for Hsa_circ_00 26782 to Inhibit Keloid Growth Both in Vitro and in Vivo

To explore the role of the interaction between hsa_circ_00 26782 and CREB1, we established hKFs overexpressing wild‐type hsa_circ_00 26782 (OE‐WT) or mutant hsa_circ_00 26782 (OE‐MUT). The EdU flow cytometry showed that, compared with the NC group, the OE‐WT group presented a lower DNA replication capacity, whereas the OE‐MUT group presented a similar ratio of EdU‐incorporated cells to the NC group (Figure 6D). Flow cytometry analysis of the cell cycle indicated that the OE‐WT arrested the cell cycle at S phase, whereas the OE‐MUT had little effect on each phase of cell cycle, compared with the NC group (Figure 6E). Next, equal amounts of NC, OE‐WT, and OE‐MUT hKFs were subcutaneously injected into nude mice. The volume of xenograft tumors followed the order of OE‐WT < NC = OE‐MUT (Figure 6F).

Taken together, suggested that, in vivo, the 1–90 nt region of hsa_circ_00 26782 bound to CREB1 and regulated keloid progression through the hsa_circ_00 26782/CREB1 axis. These results suggested that the interaction between hsa_circ_00 26782 and CREB1 was required for hsa_circ_00 26782 to inhibit the proliferation of keloids in vitro and in vivo.

### 2.8 Hydrogen Bonds Also Contribute to the Hsa_circ_**00** **26782**/CREB1 Binding

**Figure 7:**
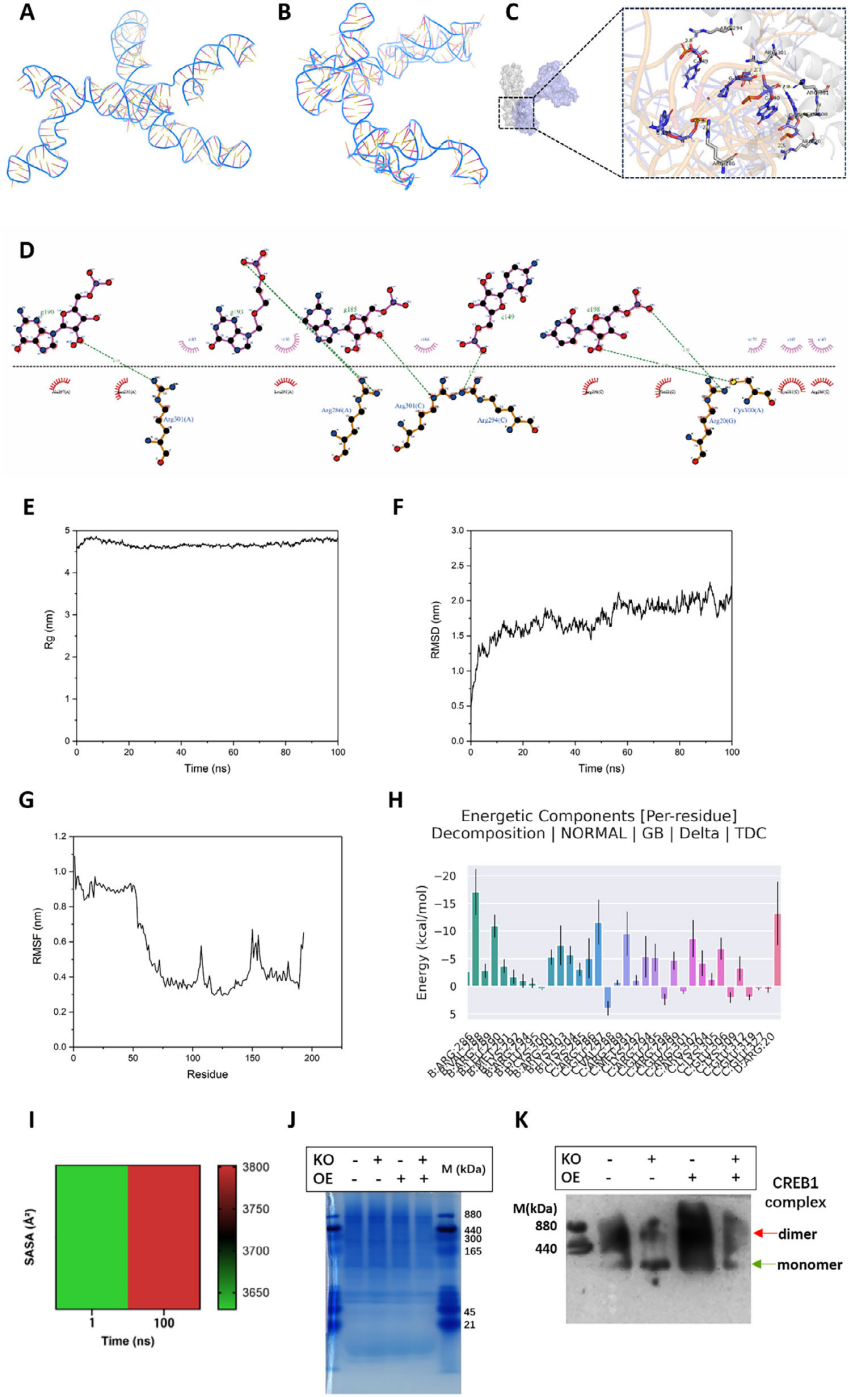
The interaction between hsa_circ_00 26782 and CREB1 increases the exposure of the bZIP domain and the dimerization of CREB1. (A) 3D schematic of the linearized hsa_circ_00 26782 tertiary structure. “C” denotes cytosine and “G” denotes guanine, with numbers representing their positions within the RNA sequence; “ARG” denotes arginine. (B) 3D schematic of the circular hsa_circ_00 26782 tertiary structure. (C) 3D schematic diagram of hsa_circ_0026782‐CREB1 binding. The RNA and protein were shown as cartoon models. “N” atoms were shown in blue, and “O” atoms in red. (D) 2D diagram of hsa_circ_0026782‐CREB1 binding. The hsa_circ_00 26782 was shown as a purple ball‐and‐stick model, and the CREB1 protein was shown as a brown ball‐and‐stick model. The green dashed line indicated the hydrogen bonds formation. Carbon atoms were shown in black, and oxygen atoms were shown in red. (E) Radius of gyration (Rg) in a 100 ns MD simulation. The Rg value was used to evaluate the closeness of the architecture. A larger value indicated a looser protein, and a smaller value indicated a tighter protein. The Y‐axis represented the Rg value, and the X‐axis represented the MD simulation time. (F) Root mean square deviation (RMSD) in the 100 ns MD simulation. The RMSD value was used to observe the overall protein conformational changes of the system relative to the initial structure during the simulation. A higher value indicated a greater structural change of the protein in the system. The Y‐axis represented the RMSD value, and the X‐axis represented the MD simulation time. (G) Root mean square function (RMSF) of 193 modelled remaining residues in the 100 ns MD simulation. A larger value suggested greater fluctuation of residues. The Y‐axis represented the RMSF value, and the X‐axis represented the number of residues. (H) Residue decomposition plot of hsa_circ_0026782‐CREB1 in 100 ns MD simulations. The Y‐axis represented the energy value, and the X‐axis represented the position and name of residues (chain: residue abbreviation: residue number). (I) Heatmap of SASA value in 100 ns molecular dynamic simulation trajectory. (J) Coomassie brilliant blue staining of SDS‐PAGE gel for native western blot. “M” represented the nondenaturing protein marker. (K) CREB1 dimer complex in total hKFs was detected by native western blot. Coomassie brilliant blue staining was used as an internal control. All experiments were performed in triplicate. Error bars indicate the mean ± SD. ns *p* > 0.05, *p < 0.05, **p < 0.01, ***p < 0.001 by independent t‐tests.

On the basis of above results, we speculated that the higher structure of hsa_circ_00 26782, along with its non‐covalent binding with CREB1, might play an indispensable role in the binding process. Therefore, we predicted the secondary structures of the hsa_circ_00 26782 truncations using RNAfold. The prediction results revealed that the secondary structures of 1–90 nt (Figure S8A, Supporting Information) and 1–222 nt (Figure S8C, Supporting Information) were similar to that of full‐length hsa_circ_00 26782, whereas the secondary structure of 1–127 nt (Figure S8B, Supporting Information) differed obviously from that of full‐length hsa_circ_00 26782. Subsequently, we used the 3dRNA webserver to predict the tertiary structures of the three truncations and the linearized hsa_circ_00 26782 (**Figure** **7**A). Compared with the full‐length linearized hsa_circ_00 26782, the tertiary structures of the 1–127 nt (Figure S8E, Supporting Information) and 1–222 nt (Figure S8F, Supporting Information) were substantially different, whereas the 1–90 nt (Figure S8D, Supporting Information), owing to its short length, had a much more similar tertiary structure. The predictions of the secondary structures and linearized tertiary structures supported our hypothesis that the higher structure played a crucial role in the binding of hsa_circ_00 26782 to CREB1.

To investigate whether there were the non‐covalent interactions between hsa_circ_00 26782 and CREB1, we predicted the tertiary structure of circular hsa_circ_00 26782 using 3dRNA webserver and visualized it with PyMOL (Figure 7B). Subsequently, we performed molecular docking simulations between the predicted tertiary structure of circular hsa_circ_00 26782 and the crystal structure of CREB1 homodimer (PDB ID: 5ZKO). On basis of the low binding free energy principle, we selected the top‐scoring conformation, with a docking affinity score of ‐490.19, this negative value indicated that the hsa_circ_00 26782 and CREB1 could spontaneously combine. In this conformation, the 3D schematic was constructed (Figure 7C), and indicated that specific nucleotide residues of hsa_circ_00 26782 were engaged in non‐covalent interactions (mainly through hydrogen bonds) with specific amino acid residues of CREB1. Specifically, hydrogen bonds were formed between the G190 base of the RNA and the ARG301 residue of chain A; the G193 base and the ARG286 residue of chain A; the G185 base and the ARG301 residue of chain C; the C149 base and the ARG301 residue of chain C; and the C198 base, which formed hydrogen bonds with the ARG20 residue of chain G and the CYS300 residue of chain A (“G” stands for guanine and “C” stands for cytosine, with numbers representing their positions within the RNA sequence; “ARG” stands for arginine and “CYS” stands for cysteine; Figure 7D). However, the KID region was absent within the crystal structure of CREB1, which prevented us from analyzing the non‐covalent bonds between KID and hsa_circ_00 26782 and the conformational changes in the KID region after the combination of hsa_circ_00 26782 and CREB1.

### 2.9 The Interaction Between Hsa_circ_00 26782 and CREB1 Increases the Exposure of the bZIP Domain and the Dimerization of CREB1

In order to explore the possible conformational changes caused by the binding of hsa_circ_00 26782 and CREB1, Gromacs2021 software was applied to do molecular dynamics (MD) simulation. The radius of gyration (Rg) is commonly used to evaluate the compactness of a molecular complex, a larger Rg value indicates a more extended or less compact protein structure. When the Rg value exceeds 4 nm, it generally suggests a relatively loose interaction. Our MD simulation showed that during the 100 ns simulation, the Rg values of the hsa_circ_0026782‐CREB1 complex ranged between 4.54923 and 4.86 459 nm, with relatively small fluctuations, and at 100 ns the Rg value was 4.81 427 nm (Figure 7E). The result indicated the interaction between hsa_circ_00 26782 and the CREB1 homodimer (excluding the KID domain) was relatively loose, which also suggested that the covalent binding interface between hsa_circ_00 26782 and CREB1 may not be present in the currently available X‐ray crystallographic structure of CREB1.

The root mean square deviation (RMSD) is employed to observe the overall conformational changes in the protein relative to the initial structure during the simulation, and larger RMSD values indicate greater structural changes. In our study, the RMSD of the complex exhibited minor fluctuations during the first 20 ns, but as the simulation progressed, the fluctuations diminished, and the RMSD stabilized at 2.28 477 nm (Figure 7F). The RMSD exceeding 0.5 nm typically suggests substantial structural alterations within the protein system, which may involve changes in the overall folding state, rearrangement of domains, significant disruption of secondary structures, or a transition between different functional states of the protein. Our RMSD value suggested that the binding of hsa_circ_00 26782 to the CREB1 might induce pronounced structural changes or fundamental alterations in the function of CREB1.

The root mean square fluctuation (RMSF) is used to evaluate the local structural flexibility of amino acid residues during the simulation, and higher RMSF values indicate greater flexibility. The RMSF value greater than 0.3 nm indicates that the corresponding amino acid residues exhibit high flexibility. These flexible residues are usually located on the protein surface, within intrinsically disordered regions, or in proximity to the active site, and might contribute significantly to protein‐protein interactions, ligand binding, and signal transduction processes. Our analysis revealed significant fluctuations in the protein residues, with residues 1–60 showing much greater fluctuations than the other residues did (Figure 7G).

According to the principle of minimum free energy, a residue decomposition plot was performed to indicate the interaction between hsa_circ_00 26782 and CREB1. In contrast to RMSF, a higher negative average binding energy represents a greater stabilizing contribution of the corresponding residue to the binding complex. To be detail, the MD simulation results revealed that VAL288 and LEU290 residues in chain B of the CREB1 homodimer, GLU287 and MET291 residues in chain C, and ARG20 in chain D, were found to play key roles in stabilizing the interaction between hsa_circ_00 26782 and CREB1, contributing more significantly to the binding than other residues did (“VAL” stands for valine, “LEU” stands for leucine, “GLU” stands for glutamic, “MET” stands for methionine, and “ARG” stands for arginine; Figure 7H). In summary, the hsa_circ_0026782‐CREB1 complex exhibited stable binding.

Next, we used the solvent‐accessible surface area (SASA) to determine the conformational change in the bZIP region (amino acid sequence: LENRVAVLENQNKTLIEELKAL). SASA refers to the surface area of a biomolecule that can be contacted by a solvent, and the SASA of bZIP domain is used to represent the exposure degree in this study. In the initial structure without RNA binding, the bZIP domain had a SASA of 3630.15 Å^2^; whereas in the final frame of the molecular dynamic simulation trajectory, the SASA of the bZIP domain with RNA binding increased to 3802.11 Å^2^ (Figure 7I), which indicated that the exposure of bZIP domain might increase after the binding of hsa_circ_00 26782 with CREB1.

As a member of the bZIP family, CREB1 typically exists as a dimer through its bZIP domain. The MD simulations indicated that the interaction between hsa_circ_00 26782 and CREB1 might lead to increased exposure of the bZIP domain. To determine whether hsa_circ_00 26782 affected CREB1 dimer formation, we extracted total proteins from hKFs by nondenaturing lysis buffer and performed native western blot analysis. Coomassie blue staining of the SDS‐PAGE gel was used as loading control (Figure 7J), and a CREB1 antibody was used for detection of CREB1 dimer complex. The results showed that the ratio of the CREB1 dimer (upper band) to monomer (lower band) increased when hsa_circ_00 26782 was overexpressed, whereas the ratio decreased when hsa_circ_00 26782 was knocked out (Figure 7K). Our results suggested that hsa_circ_00 26782 binding might enhance CREB1 dimer formation.

### 2.10 Hsa_circ_00 26782 Inhibits Keloid Growth by Promoting CREB1 Phosphorylation at Ser142

**Figure 8:**
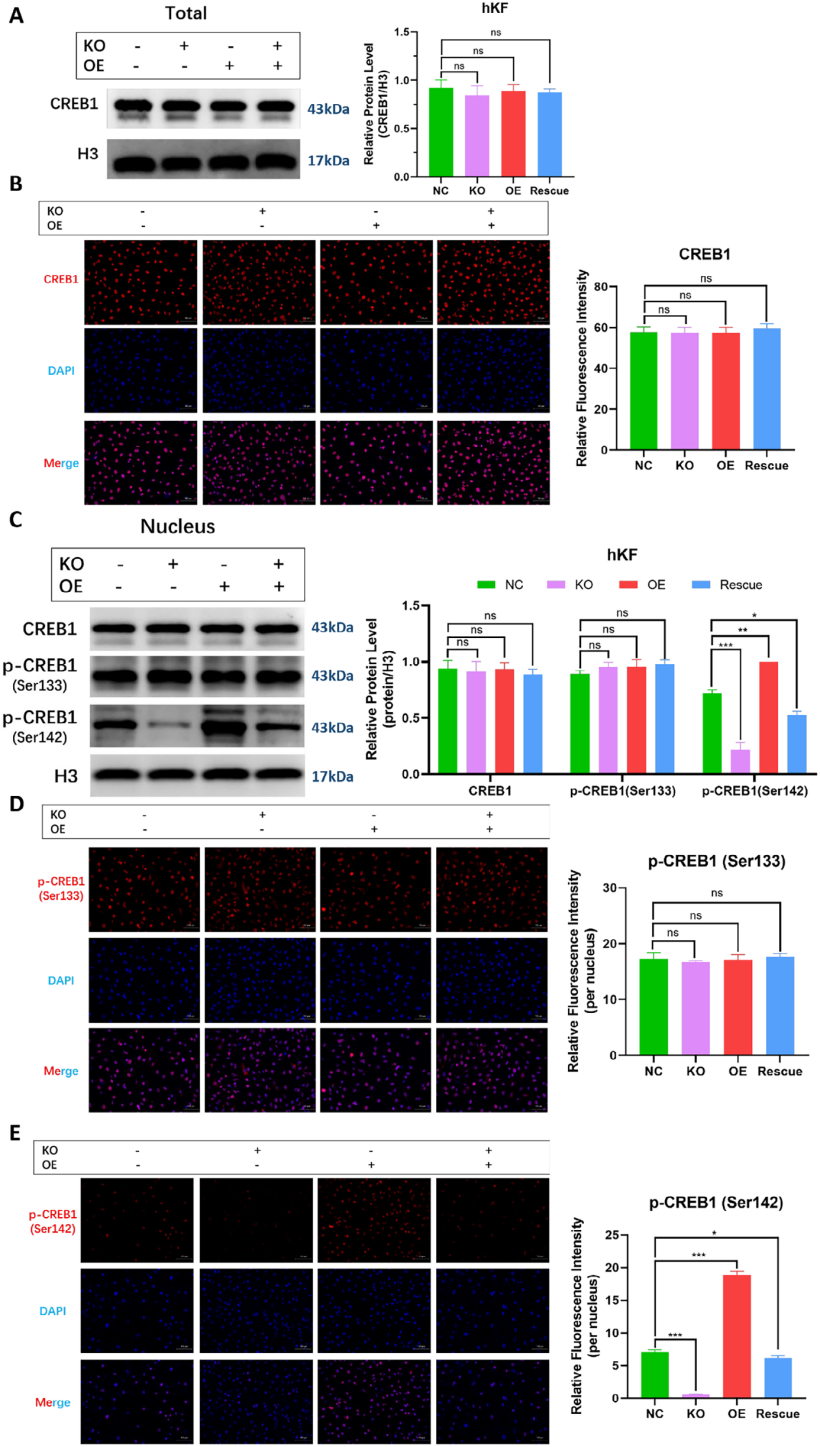
Hsa_circ_00 26782 inhibits keloid growth by promoting CREB1 phosphorylation at Ser142. (A) CREB1 protein level in total hKFs was detected by western blot (left), and the quantification of relative protein levels (right) were shown. Histone 3 (H3) was used as an internal control. (B) Immunofluorescence staining of CREB1 (red) in hKFs (left), and the quantification of relative fluorescence intensities (right) were shown. Cell nuclei were stained with 4,6‐diamidino‐2‐phenylindole (DAPI; blue). Original magnification: 200×. (C) CREB1, p‐CREB1 (Ser133) and p‐CREB1 (Ser142) protein levels in nucleus of hKFs was detected by western blot (left), and the quantification of relative protein levels (right) were shown. (D) Immunofluorescence staining of p‐CREB1 (Ser133) (red) in hKFs (left), and the quantification of relative fluorescence intensities (right) were shown. (E) Immunofluorescence staining of p‐CREB1 (Ser142) (red) in hKFs (left), and the quantification of relative fluorescence intensities (right) were shown. All experiments were performed in triplicate. Error bars indicate the mean ± SD. ns *p* > 0.05, *p < 0.05, **p < 0.01, ***p < 0.001 by independent t‐tests.

To further verify whether hsa_circ_00 26782 directly regulated the protein stability of CREB1, we examined the total protein levels of CREB1 in hKFs. The western blot results revealed that hsa_circ_00 26782 did not affect the level of CREB1 protein (**Figure** **8**A), and the immunofluorescence staining also showed that CREB1 signal intensity was similar in NC, KO, OE, and Rescue groups (Figure 8B). These results indicated that hsa_circ_00 26782 had little effect on the protein stability of CREB1. CREB1 is synthesized by ribosomes in the cytoplasm, and then transported into the nucleus waiting for phosphorylation. We extracted nuclear proteins from hKFs (Figure S9, Supporting Information) and examined the nuclear translocation of CREB1. The results showed that hsa_circ_00 26782 did not affect CREB1 nuclear translocation (Figure 8C).

Notably, the transactivation of CREB1 is mainly regulated by its phosphorylation status. To explore whether hsa_circ_00 26782 affected CREB1 phosphorylation, we measured the phosphorylation status at two sites: Ser133, which enhances transactivation of CREB1 (p‐CREB1 (Ser133)); and Ser142, which inhibits CREB1 transcriptional activity (p‐CREB1 (Ser142)). The western blot results showed that the level of p‐CREB1 (Ser133) remained similar regardless of hsa_circ_00 26782 expression (whether overexpressed, knocked out, or rescued). However, the level of p‐CREB1 (Ser142) obviously increased with hsa_circ_00 26782 overexpression and decreased with hsa_circ_00 26782 knockout (Figure 8C). The results were confirmed by immunofluorescence. No obvious change of p‐CREB1 (Ser133) fluorescence intensity in nuclei was observed in different groups (Figure 8D), whereas the fluorescence intensity of p‐CREB1 (Ser142) in nuclei was markedly increased in hsa_circ_00 26782 OE group and dramatically decreased in KO group (Figure 8E). These findings demonstrated that hsa_circ_00 26782 significantly increased p‐Ser142 in CREB1 without affecting p‐Ser133 levels.

**Figure 9:**
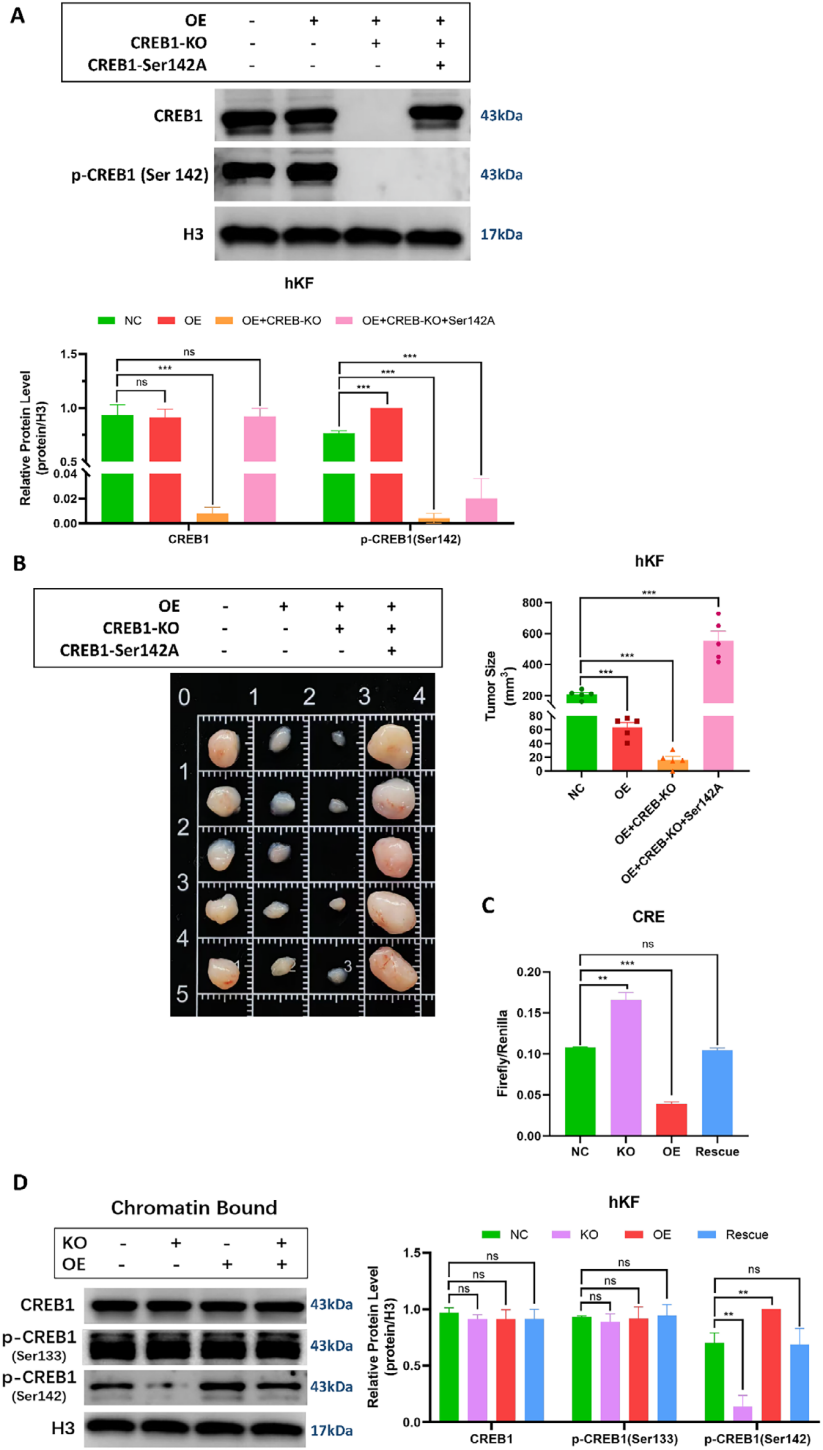
Hsa_circ_00 26782 inhibits keloid growth by promoting CREB1 phosphorylation at Ser142. (A) Knockout efficiency of endogenous CREB1 (CREB1‐KO) and overexpression efficiency of the exogenous residue 142 mutant CREB1 (CREB1‐Ser142A) were measured via western blot (upper), and the quantification of relative protein levels (lower) were shown. H3 was used as an internal control. (B) Six‐week‐old female BALB/c nude mice were subcutaneously injected with 1 × 10^7^ of the indicated hKFs. Animals were sacrificed at week eight, and the xenografts were isolated (left) and measured (right). (C) The transcription level of exogenous CRE in hKFs was detected by a dual‐luciferase reporter assay. The luciferase signal was normalized by Firefly/Renilla. (D) Chromatin‐bound protein levels of CREB1, p‐CREB1 (Ser133), and p‐CREB1 (Ser142) (left), and the quantification of relative protein levels (right) were shown. H3 was used as an internal control. All experiments were performed in triplicate. Error bars indicate the mean ± SD. ns *p* > 0.05, **p < 0.01, ***p < 0.001 by independent t‐tests.

To explore whether hsa_circ_00 26782 inhibited keloid growth in vivo by promoting p‐Ser142 of CREB1, we generated CREB1‐Ser142A non‐phosphorylated mutant^[^ ^27^, ^55^ ^]^ in hsa_circ_0026782‐overexpression (OE) and CREB1 knockout (CREB1‐KO) hKFs (**Figure** **9**A). To avoid unexpected cellular effects caused by different levels of CREB1 expression, cells with the expression level of mutant CREB1‐Ser142A similar to the level of endogenous CREB1 were selected and cultured for further experiments. Equal amounts of untreated (NC), OE, OE+CREB1‐KO, or OE+CREB‐KO+CREB1‐Ser142A hKFs, were subcutaneously injected into nude mice. The results of the tumor‐bearing experiments showed that the tumor volumes of the xenografts decreased in the following order: OE+CREB1‐KO < OE < NC < OE+CREB‐KO+CREB1‐Ser142A. The results demonstrated that hsa_circ_00 26782 could inhibit keloid progression driven by wildtype CREB1, but could not inhibit keloid progression driven by CREB1‐Ser142A non‐phosphorylated mutant (Figure 9B).

In addition to serving as the dimerization site for CREB1, the bZIP domain is also the main region for CREB1 to bind to DNA. The DNA‐binding ability of CREB1 is manifested in the fact that CREB1 can recognize specific DNA sequences—CRE, through its bZIP domain. We performed a dual‐luciferase reporter assay by co‐transfecting hKFs with the firefly luciferase reporter plasmid pCREB‐TA‐Luc, which contained five exogenous target gene CRE sequences, and the renilla luciferase reporter plasmid pRL‐SV40‐N. The results showed that when hsa_circ_00 26782 was knocked out, the luciferase signal was significantly enhanced, whereas the overexpression of hsa_circ_00 26782 led to a low luciferase signal (Figure 9C), indicating the DNA binding capacity of CREB1 was inhibited by hsa_circ_00 26782. Based on SASA analysis of the bZIP domain, binding of hsa_circ_00 26782 to CREB1 increased the exposure of bZIP domain, which may also increase the binding of CREB1 to CRE sequence. However, our results showed that overexpression of hsa_circ_00 26782 actually reduced the ratio of Firefly/Renilla luciferase. We hypothesized that this may result from the low transactivation of CREB1 induced by hsa_circ_00 26782. The chromatin binding ability of CREB1 was further investigated by extracting chromatin‐bound proteins and assessing the protein levels of chromatin‐bound CREB1, p‐CREB1 (Ser133), and p‐CREB1 (Ser142). The western blot results showed that the overexpression of hsa_circ_00 26782 obviously increased the level of chromatin‐bound p‐CREB1 (Ser142); in contrast, when hsa_circ_00 26782 was knocked out, only a minimal amount of chromatin bound p‐CREB1 (Ser142) was observed (Figure 9D). Interestingly, the levels of chromatin‐bound CREB1 and p‐CREB1 (Ser133) remained unchanged, suggested that hsa_circ_00 26782 did not influence the binding between CREB1 and chromatin.

### 2.11 Hsa_circ_00 26782 Inhibits the Transcriptional Activity of CREB1

The phosphorylation of Ser133 turned CREB1 into high transcriptional activity. However, p‐Ser142 was reported to have the opposite effect, leading to CREB1 with lower transcriptional activity. To investigate the influence of hsa_circ_00 26782 on the CREB1‐mediated transcriptions, we conducted whole‐transcriptome sequencing on hKFs overexpressing hsa_circ_00 26782 (OE) or with CREB1 knocked out (CREB1‐KO), compared with untreated hKFs (negative control, NC), to investigate the downstream effectors of the hsa_circ_00 26782/CREB1 axis.

**Figure 10:**
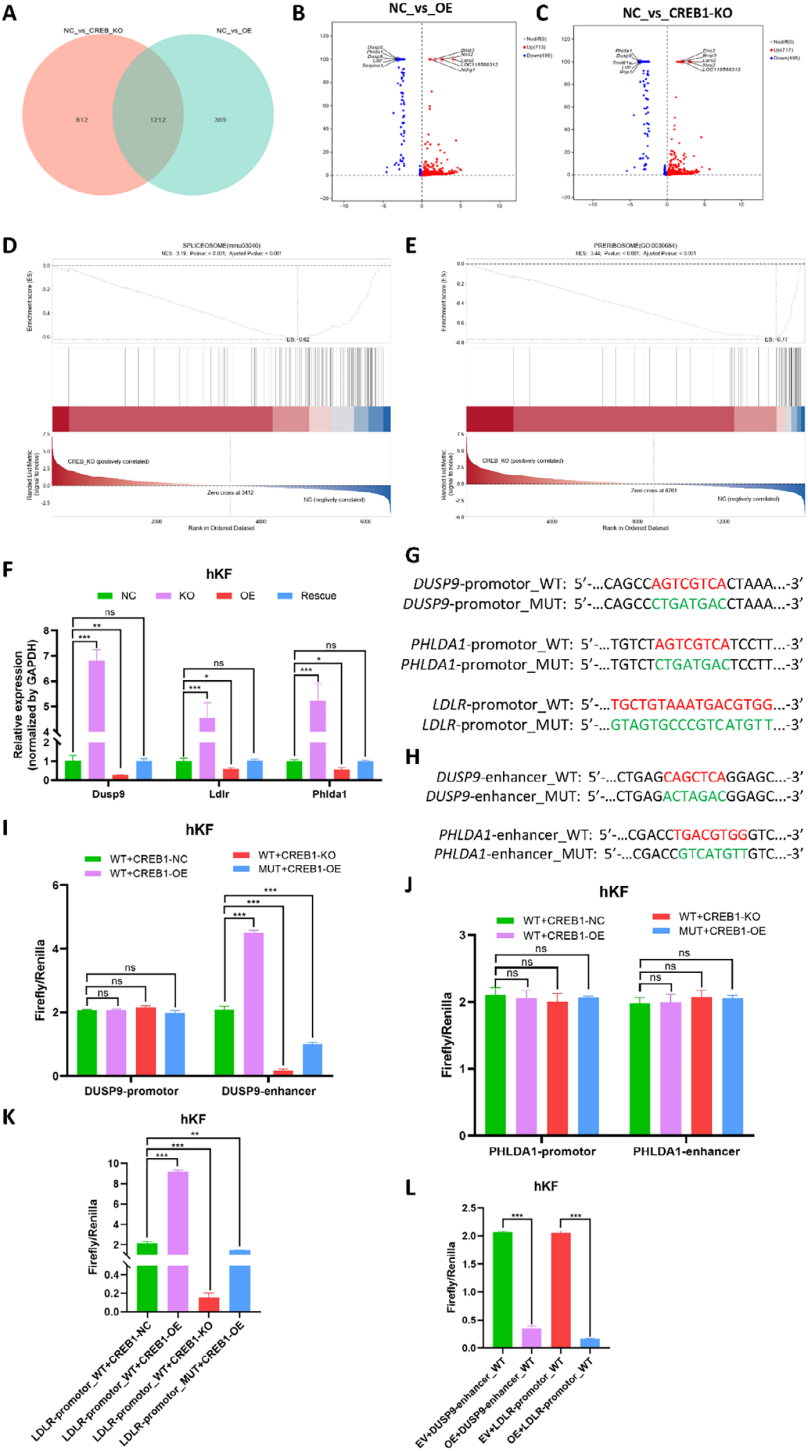
The Ser142 mutation in CREB1 can reverse the biological effects mediated by hsa_circ_00 26782. (A) Venn diagrams of DEGs between NC group and OE group (orange) or NC group and CREB1‐KO group (green), and the number of DEGs were indicated. (B‐C) Volcano plot of DEGs between NC group and OE group (B) or NC group and CREB1‐KO group (C). Each plot represented a DEG. Upregulated DEGs were marked in red, downregulated DEGs were marked in blue, and the gray plot represented non‐differentially expressed genes. (D) GSEA for the KEGG spliceosome pathway (mmu03040) of hKFs with CREB1 knockout, compared to normal hKFs. (E) GSEA for the GO preribosome gene set (GO:00 30684) of hKFs with CREB1 knockout, compared to normal hKFs. (F) The endogenous transcription level of three downregulated DEGs (Dusp9, Ldlr and Phlda1) in hKFs with different has_circ_00 26782 expression levels were detected by RT‐qPCR. (G) Predicted potential promoter‐binding sites of *DUSP9* and *PHLDA1* for CREB1, and the SIRE sequence within the *LDLR* promoter predicted to interact with CREB1, along with their corresponding mutant sequences. (H) Predicted potential enhancer‐binding sites of *DUSP9* and *PHLDA1* for CREB1, along with their corresponding mutant sequences. (I) Dual‐luciferase reporter assays showed the interaction between CREB1 and both the promoter and enhancer regions of *DUSP9* (including wild‐type and mutant constructs). The relative luciferase activity was presented as firefly/renilla. (J) Validation of the interactions between CREB1 and the promoter or enhancer of *PHLDA1* by dual‐luciferase reporter assay. (K) Validation of the interactions between CREB1 and the promoter of *LDLR* by dual‐luciferase reporter assay. (L) Effects of hsa_circ_00 26782 on CREB1‐mediated transcription of *DUSP9* and *LDLR* were measured by dual‐luciferase reporter assays, the relative luciferase activity was shown as firefly/renilla. All experiments were performed in triplicate. Error bars indicate the mean ± SD. ns *p* > 0.05, *p <  0.05, **p <  0.01, ***p < 0.001 by independent t‐tests.

Differentially expressed genes (DEGs) were identified by comparing each treatment group with the NC group. Intersection analysis revealed a total of 1212 DEGs that were commonly dysregulated in both comparisons (**Figure** **10**A). Specifically, in the OE versus NC comparison, 713 genes were upregulated and 499 were downregulated (Figure 10B); while in the CREB1‐KO versus NC comparison, 717 genes were upregulated and 495 were downregulated (Figure 10C). Subsequently, we conducted Gene Set Enrichment Analysis (GSEA) based on the RNA‐seq data from CREB1‐KO and NC groups. The spliceosome pathway (KEGG mmu03040) was significantly negatively enriched in CREB1‐deficient cells (NES = ‐3.19, adjusted *p*‐value< 0.001), indicating downregulation of genes involved in mRNA splicing (Figure 10D). In addition, the preribosome gene set (GO:00 30684) showed an even stronger negative enrichment (NES = ‐3.44, adjusted *p*‐value<0.001), suggesting substantial repression of genes involved in ribosome biogenesis (Figure 10E). These findings imply that CREB plays a critical role in maintaining the expression of genes involved in both post‐transcriptional processing and ribosomal assembly, and its loss may impair global mRNA metabolism and protein synthesis capacity.

Building upon the transcriptomic findings above, we next sought to identify key downstream targets of the hsa_circ_00 26782/CREB1 axis. Based on the GSEA results, we focused subsequent analyses on the downregulated DEGs. We compared the top five most downregulated genes in both the OE versus NC and CREB1‐KO versus NC groups, and identified three overlapping DEGs: dual specificity phosphatase 9 (Dusp9), low density lipoprotein receptor (Ldlr), and pleckstrin homology like domain family A member 1 (Phlda1), that were consistently and significantly downregulated in both comparisons. To further validate this finding, we examined whether the expression levels of these genes were altered under different levels of hsa_circ_00 26782 expression. The results demonstrated that knockout of hsa_circ_00 26782 markedly increased the expression levels of Dusp9, Ldlr, and Phlda1, while overexpression of hsa_circ_00 26782 led to their downregulation (Figure 10F). These findings suggest that the hsa_circ_00 26782/CREB1 axis regulates a broad set of target genes and may function as a “central regulatory hub” in the biological processes of fibroblasts and keloid pathogenesis.

After verifying that *DUSP9*, *LDLR*, and *PHLDA1* are downstream targets of the hsa_circ_00 26782/CREB1 axis, we next aimed to confirm whether CREB1 directly binds to the promoter regions of these genes. A previous study utilizing chromatin immunoprecipitation followed by sequencing (ChIP‐seq) in HEK‐293T cells identified *DUSP9* and *LDLR* as potential CREB1 binding targets, whereas *PHLDA1* was not detected.^[^ ^56^ ^]^ In addition, Jingwen Liu et al. reported that although the *LDLR* promoter lacks a canonical CRE sequence, a sterol‐independent regulatory element (SIRE) located 17 bp upstream of the transcription start site can interact with CREB1.^[^ ^57^ ^]^ To further assess the direct binding of CREB1 to the promoters of *DUSP9*, *LDLR*, and *PHLDA1*, we constructed dual‐luciferase reporter plasmids by using pmirGLO plasmid, which containing both firefly and renilla luciferase genes. For *LDLR*, we generated a wild‐type promoter construct containing the intact SIRE sequence (*LDLR*‐promoter_WT) and a mutant construct in which the SIRE sequence was disrupted by substituting “A” with “C” and “T” with “G” (*LDLR*‐promoter_MUT; Figure 10G). For *DUSP9* and *PHLDA1*, we first identified their genomic loci using the UCSC Genome Browser in combination with the ENCODE Project database (https://www.encodeproject.org). The 2,000 bp upstream of each gene was defined as the putative promoter region. Canonical CRE sequences were not detected in either promoter, suggesting that CREB1 might bind to variant CRE sequences or the enhancers. Therefore, we performed motif scanning using the position weight matrix (PWM) of CREB1 to predict potential binding sites within the promoter sequences. The analysis revealed a putative low‐conservation CREB1 binding motif (sequence: AGTCGTCA) in both *DUSP9* and *PHLDA1* promoters. Based on these findings, we constructed wild‐type and mutant dual‐luciferase reporter plasmids for each gene (*DUSP9‐*promoter_WT, *DUSP9*‐promoter_MUT, *PHLDA1*‐promoter_WT, and *PHLDA1*‐promoter_MUT; Figure 10G). Next, we explored potential enhancer elements of *DUSP9* and *PHLDA1* using the GeneHancer database (https://genecards.org/GeneHancer/). The prediction identified three enhancers for *DUSP9* and five for *PHLDA1*. Among them, the highest‐scoring enhancer of *PHLDA1* contained a canonical CRE motif. We subsequently constructed *PHLDA1*‐enhancer_WT and *PHLDA1*‐enhancer_MUT reporter plasmids targeting this CRE site. In contrast, none of the predicted enhancers of *DUSP9* contained canonical CRE motifs. Thus, we applied CREB1 PWM scanning again to predict potential binding sites within the *DUSP9* enhancer sequence and generated corresponding wild‐type and mutant reporter constructs (*DUSP9*‐enhancer_WT and *DUSP9*‐enhancer_MUT; Figure 10H).

By co‐transfecting hKFs with dual‐luciferase reporter plasmids containing either wild‐type or mutant promoter/enhancer sequences, along with CREB1 overexpression (CREB1‐OE) or knockout (CREB1‐KO) plasmids, we assessed the transcriptional regulation of target genes. We observed that luciferase signals of the two *DUSP9* promoter groups remained unchanged regardless of CREB1 expression levels. In contrast, the *DUSP9*‐enhancer_WT group exhibited a significant increase in luciferase activity following CREB1 overexpression, whereas the *DUSP9*‐enhancer_MUT group showed reduced signal. Furthermore, luciferase activity of the *DUSP9‐*enhancer_WT group was markedly decreased upon CREB1 knocked out (Figure 10I). These results suggested that CREB1 directly binds to the *DUSP9* enhancer to regulate its transcription. Notably, luciferase signals from both wild‐type and mutant *PHLDA1* promoter and enhancer groups were unaffected by changes in CREB1 expression (Figure 10J), indicating that CREB1 does not directly bind to the promoter or enhancer of *PHLDA1*. We speculated that CREB1 may regulate *PHLDA1* transcription indirectly through alternative signaling pathways or transcriptional networks. In addition, overexpression of CREB1 significantly enhanced luciferase activity in the *LDLR*‐promoter_WT group, while the signal was reduced in the *LDLR*‐promoter_MUT group, and CREB1 knockout resulted in the lowest luciferase activity across all groups (Figure 10K). These findings confirmed that CREB1 regulates *LDLR* transcription by binding to the SIRE element within its promoter. Finally, we co‐transfected hKFs with hsa_circ_00 26782 overexpression (OE) or empty vector (EV) plasmids along with *LDLR*‐promoter_WT or *DUSP9*‐enhancer_WT plasmids. Compared with the EV group, overexpression of hsa_circ_00 26782 significantly suppressed luciferase activity from both *LDLR* and *DUSP9* (Figure 10L), further supporting that hsa_circ_00 26782 inhibited CREB1‐mediated transcription of downstream genes, thereby contributing to the suppression of keloids.

## 3 Discussion

Although significant progress has been made in the study of circRNAs across various diseases, revealing numerous novel mechanisms, researches on circRNAs in keloids remains relatively scarce. Keloids are highly recurrent conditions that cause considerable psychological and physical distress to patients. Unfortunately, treatments are unable to fully resolve keloids yet, and can alleviate only symptoms and side effects to a limited extent. The pain from repeated recurrences, coupled with the treatments themselves can have a devastating impact on the well‐being of patients. To address the present dilemma, our aim is to address the root causes of keloid formation and recurrence while minimizing patient suffering. In this study, focusing on the characteristics of keloids, namely, genetic heterogeneity and individual specificity, we conducted high‐throughput sequencing of keloid tissues and normal scar tissues and identified a novel circRNA, hsa_circ_00 26782. Hsa_circ_00 26782 is downregulated in keloids and can significantly inhibit keloid formation and progression both in vitro and in vivo.

Recent studies have suggested that the upregulation of proinflammatory factors in keloids indicates that keloids are not skin tumors but rather inflammatory skin diseases, particularly involving inflammation of the reticular dermis,^[^ ^10^ ^]^ and defined keloids as highly inflamed pathological scars. However, numerous studies have confirmed that keloids exhibit tumor‐like characteristics, such as excessive proliferation and invasion of normal tissues. CREB1, a known oncogenic factor, has been implicated in several cancers, including colorectal cancer,^[^ ^58^ ^]^ cervical cancer,^[^ ^59^ ^]^ liver cancer,^[^ ^60^ ^]^ lung cancer,^[^ ^61^ ^]^ and so on. Moreover, CREB1 is closely associated with skin inflammation. Jiaoling Chen et al. reported that CREB1‐driven CXCR4(high) neutrophils promote skin inflammation in both mouse models and human patients.^[^ ^62^ ^]^ Our study reveals that the hsa_circ_00 26782/CREB1 axis may play a crucial role in the progression of keloids, providing strong evidence supporting the notion that “keloids are a kind of inflammatory skin disease with tumor‐like properties”. Moreover, the three significantly downregulated DEGs downstream of the hsa_circ_00 26782/CREB1 axis (*DUSP9*, *LDLR*, and *PHLDA1*) have been reported in multiple studies to be closely associated with various types of cancer,^[^ ^63^, ^64^, ^65^, ^66^, ^67^ ^]^ inflammation,^[^ ^66^, ^68^, ^69^ ^]^ and fibrosis.^[^ ^70^, ^71^ ^]^ These findings further support the notion that keloids exhibit tumor‐like properties, excessive inflammation, and fibrotic features, and highlight the biological relevance of hsa_circ_00 26782 both in vitro and in vivo. Notably, these three DEGs have not yet been explored in the context of keloid pathogenesis, suggesting promising directions for future research.

Here, we have also demonstrated that the 1–90 nt region of hsa_circ_00 26782 binds to CREB1, leading to an increased exposure of the bZIP domain of CREB1, which in turn promotes CREB1 dimerization and enhanced its DNA‐binding capacity. This seems contradictory to the conclusion that “hsa_circ_00 26782 inhibits keloids both in vitro and in vivo”, for reason of CREB1 is a transcription factor known to promote cell tumorigenesis and tissue inflammation. Further investigation uncovers that hsa_circ_00 26782 promotes the phosphorylation of CREB1 at Ser142 (p‐Ser142). It is well known that phosphorylation at Ser133 (p‐Ser133) promotes the expression of CREB1 target genes. In contrast, p‐Ser142 of CREB1 results in transcriptional stalling to downregulate the expression of CREB1 target genes. Some studies have also suggested that the two phosphorylation events at Ser133 and Ser142 occur simultaneously, and that p‐Ser142 provides precise regulation of CREB1 target gene transcription.^[^ ^27^ ^]^ Our study has demonstrated that hsa_circ_00 26782 has little effect on the protein expression or nuclear translocation of CREB1, the chromatin binding of CREB1 or the chromatin binding of p‐Ser133 CREB1. However, the p‐Ser142 level of chromatin‐bound CREB1 is significantly increased. Our findings support the hypothesis that p‐Ser142 occurs in addition to p‐Ser133 phosphorylation, and prematurely terminating the transcription of its downstream target genes, thereby regulating transcription in a precise spatiotemporal manner.

In conclusion, our study presents a novel finding: hsa_circ_00 26782 is downregulated in keloid tissues; it acts as a “molecular break” to inhibit keloid growth by promoting the phosphorylation of CREB1 at Ser142 to enhance CREB1 dimerization, which increases CREB1 DNA‐binding ability. Thus, low‐transactivating form of p‐Ser142 CREB1 competes with highly transactivating CREB1 to bind to target DNAs. Consequently, low transactivation leads to the downregulation of expression of procarcinogenic and proinflammatory genes, ultimately inhibiting keloid growth. Due to their closed‐loop structure, circRNAs are resistant to RNase degradation and exhibit a relatively long half‐life. We believe that hsa_circ_00 26782 holds great potential as an RNA‐based therapeutic for keloids. Given the universal role of CREB1, it can potentially be applied to other tumorigenic and inflammatory diseases.

## 4 Experimental Section

### Ethical Declaration

This study was approved by the Ethics Review Committee of Sir Run Run Shaw Hospital of Zhejiang University (No. 2024‐2595‐01), followed the tenets of the Declaration of Helsinki. Written informed consent was obtained from each donor.

### Sample Acquisition

The human keloid tissues were derived from the keloid sample bank of our department, and were collected from the patients who did not accept other non‐surgical treatments (such as local injection of corticosteroids or chemotherapy drugs) and received keloid surgical excision at our hospital. In addition, the patients carrying other skin diseases were also excluded in this research. To be specific, fifteen keloid tissues were got from the sample bank, three keloid tissues were cut into two parts due to their huge size, and used separately for purpose of circRNA high‐throughput sequencing and the isolation of human keloid fibroblasts (hKFs). And the remaining were used for RT‐qPCR experiment, and the isolation of human keloid fibroblasts (hKFs). The median age of the donors is 35±15 years.

For the reason of high skin tension and heterogeneity of individuals are significant inducements for keloid, the removal of the normal scar on the same patients may cause secondary keloids. Thus, the normal scar tissues were obtained from other nine patients (median age 36±4 years) who received internal fixation taking out operation at our hospital. Three normal scar specimens were used for circRNA high‐throughput sequencing, and the rest tissues were utilized for RT‐qPCR experiments.

The specific information of samples and corresponding patients was listed in Table S1 (Supporting Information).

### CircRNA High‐throughput Sequencing

High‐throughput sequencing of circRNAs was performed by Beijing Genomics Institution. The specific protocols were described in the “Supplementary Methods” section of Supporting Information.

### RNase R Digestion and Genome DNA (gDNA) Extraction

RNase R action system (Sangon, Shanghai, China) was prepared according to the suggested system and the time of digestion.

A Ezup Column Animal Genomic DNA Purification Kit (Sangon, Shanghai, China) was used to extract genomic DNA (gDNA) in tissues according to the user's manual. Briefly, approximately 25 mg of animal tissue was ground into powder with liquid nitrogen and added to a 1.5 mL centrifuge tube, Buffer ACL containing Proteinase K solution was added. The mixture was incubated in water bath at 56 °C for 1 h until complete lysis. After Buffer CL and absolute ethyl alcohol were added in order, the gDNA were extracted through adsorption columns.

### RNA Preparation and Quantitative Reverse Transcription PCR (RT‐qPCR)

Total RNAs were extracted from cells or tissues by using TRIzol extraction method (BioSharp, Shanghai, China). Reverse transcription was displayed through MightyScript Plus First Strand cDNA Synthesis Master Mix (Sangon, Shanghai, China). Quantitative Reverse Transcription PCR (RT‐qPCR) was performed to validate the expression level of DEcircs using a SPARKscript II SYBR One Step qRT‐PCR Kit (SparkJade, Shandong, China) on a Bio‐Rad CFX‐96 fluorescence quantitative PCR instrument (Bio‐Rad, Hercules, CA, USA). The primers of DEcircs were designed back‐to‐back (divergent primer) or crossing the BSJ (splice junction overlapping divergent (sjod) primers), and were checked by circPrimer 2.0^[^ ^72^ ^]^ (http://www.bio‐inf.cn). The primers of ITGA7 exon4 were designed as convergent primers. The convergent primers of GAPDH and NEAT1 were purchased from Beyotime (Shanghai, China). The divergent primers of GAPDH were designed back‐to‐back. All primers were detailed in Table S2 (Supporting Information). The relative expression was normalized to GAPDH and was calculated by 2^‐ΔΔCt^ method.

### Isolation and Cultivation of Primary Human Keloid Fibroblasts (hKFs)

Twelve keloid tissues were washed with Hank's Balanced Salt Solution (Beyotime, Shanghai, China), and the epidermis and subcutaneous adipose tissue were removed immediately. Subsequently, the keloid tissues were dissected and cut into small pieces (≈2 mm^3^). The tissue pieces were then inoculated in a Petri dish and incubated for 2 h of 5% CO_2_ at 37 °C. DMEM (Gibco, Carlsbad, CA, USA) containing 10% fetal bovine serum (ExCell, Suzhou, China) and Penicillin‐Streptomycin (Yeason, Shanghai, China) was added to the dish for primary human keloid fibroblasts (hKFs) cultivation. When the cells grew to 80–90% confluences, the hKFs could be sub‐cultured. The hKFs of 3rd to 6th generations in the logarithmic growth phase were used in the subsequent experiments.

### RNA Fluorescence In Situ Hybridization (FISH)

The hKFs were seeded on sterile glass coverslips and fixed with 4% paraformaldehyde, followed by permeabilization with 0.5% Triton X‐100 for 10 min. After washing with DEPC water, hKFs were pre‐hybridized in hybridization buffer (Sangon, Shanghai, China) for 30 min at 37 °C. A Cy5‐labeled specific probe (sequence: 5′‐Cy5‐ATCGGTGTGCACAGGTCCTTCC‐3′) targeting the back‐splice junction of hsa_circ_00 26782 was synthesized by Tsingke (Beijing, China) and hybridized to the target RNA overnight at 37 °C in a humidified chamber. After hybridization, hKFs were washed three times with pre‐warmed wash buffer to remove unbound probe. Nuclei were counterstained with DAPI (Beyotime, Shanghai, China) for 5 min, and coverslips were mounted using anti‐fade mounting medium. Images were acquired using a confocal laser scanning microscope (Nikon A1 Ti, Tokyo, Japan), and the subcellular localization of fluorescence signals was analyzed with ImageJ software.

### Small Interfering RNAs, Small Guide RNAs, and Transient Transfection

The small interfering RNA (siRNA) of hsa_circ_00 60927 and hsa_circ_0 003563 were designed and synthesized by Sangon (Shanghai, China). Si‐NC, a siRNA targeting a sequence that does not exist in homo sapiens, was used as a negative control. The sequences of siRNAs were listed in Table S3 (Supporting Information).

The CRISPR/Cas9 system was utilized to knock out the expression of endogenous DEcircs in cells. sgRNAs were designed to target the Alu elements upstream and downstream of hsa_circ_00 26782 in the genome. The pX44666‐GFP‐Dual sgRNAs vector (Miaoling, Shanghai, China) was digested separately with BbSI (New England Biolabs, MA, USA) and BspQI (New England Biolabs, MA, USA) at 37 °C overnight, and the sgRNAs targeting the upstream or downstream Alu elements were inserted sequentially using T4 ligase (Beyotime, Shanghai, China) to ligate between the gRNA scaffold at 37 °C overnight. The sgRNAs were designed and verified through E‐CRISP Design (http://www.e‐crisp.org/E‐CRISP/), deepHF (http://www.DeepHF.com), and Cas‐OFFinder (http://www.rgenome.net/cas‐offinder/), the sequences of sgRNAs are listed in Table S3 (Supporting Information).

Transient transfection of siRNAs or CRISPR/Cas9 plasmids (pX44666) was performed by using Lipo8000 Transfection Reagent (Beyotime, Shanghai, China) according to the manufacturer's recommendations.

### Stable Overexpression of hKFs Establishment

The linearized circRNA sequences were obtained from circPrimer. The pLCDH‐CiR‐GFP‐SV40 vector (Miaoling, Shanghai, China), a transfer plasmid specifically designed for circRNA overexpression was selected. The empty pLCDH‐CiR vector was linearized by EcoRI (New England Biolabs, MA, USA) and BamHI (Sangon, Shanghai, China) restriction enzyme digestion at 37 °C overnight. Then the linearized circRNA sequence was inserted between the two circular frames of the linearized pLCDH‐CiR vector through seamless cloning by using DNA Assembly Mix Plus (LABLEAD, Beijing, China), the seamless cloning system was incubated at 45 °C for 15 min.

A second‐generation lentiviral system (pMD2.G, psPAX2, and corresponding transfer plasmid) was applied to HEK‐293T (ATCC, VA, USA) using Lipo293F Transfection Reagent (Beyotime, Shanghai, China).^[^ ^73^ ^]^ The virus was concentrated by LYNX6000 high speed centrifuge (ThermoScientific, MA, USA). Stable overexpression hKFs (OE) and untreated hKFs (negative control, NC) were then established through virus infection and selected by Fluorescence activated Cell Sorting (FACS), and the selected hKFs were cultured in complete DMEM medium containing puromycin (2 ug/mL) as described previously.^[^ ^74^ ^]^

### Northern Blot

Total RNA was extracted from hKFs using TRIzol method. RNA concentration and qualified by using a Nanodrop2000 spectrophotometer (ThermoScientific, MA, USA) and agarose gel electrophoresis. About 15 µg of total RNA was separated on a 2% formaldehyde‐agarose gel and transferred to a nylon membrane (Amersham, Bucks, UK) by capillary transfer in 20 × SSC buffer (Sangon, Shanghai, China) overnight. The membrane was UV‐crosslinked and pre‐hybridized in hybridization buffer (5 × SSC, 50% formamide, 5 × Denhardt's solution, 0.5% SDS, 100 µg/mL salmon sperm DNA) at 42 °C for 2 h.

The biotin‐labeled probe was designed reverse complementary across the BSJ site of hsa_circ_0 02672, and was synthesized by Tsingke Biotech Co., Ltd. (Beijing, China). The sequence of biotin‐labeled probe from 5′ to 3′ was: Biotin‐ATCGGTGTGCACAGGTCCTTCC. The probe hybridized to the membrane overnight at 42 °C. After hybridization, the membrane was washed twice in 2 × SSC, 0.1% SDS at room temperature for 15 min, followed by two washes in 0.1 × SSC, 0.1% SDS at 50 °C for 15 min. Signals were detected using a Chemiluminescent Biotin‐labeled Nucleic Acid Detection Kit (Beyotime, Shanghai, China). Ribosome RNA 18S and 28S were used as internal loading control to normalize the expression levels.

### Cell Counting Kit‐8 Assay

The Cell Counting Kit‐8 (CCK‐8) assay was displayed to assess cell proliferation using the Super‐Enhanced Cell Counting Kit‐8 (Beyotime, Shanghai, China) in accordance with the manufacturer's instructions. Briefly, approximately 2 × 10^4^ hKFs were seeded into each well of 96‐well plates. Then, 10 µL of CCK‐8 solution was added to each well and incubated at 37 °C for 1 h. The cell proliferation curves were plotted by measuring the absorbance at 450 nm at each indicated time point. Experiments were performed in triplicate.

### Wound Healing Assay

The hKFs were seeded at 3 × 10^5^ cells per well in 6‐well plates. A sterilized Culture‐Insert 2 Well (ibidi, Planegg, Germany) was used to make a straight scratch of close width in the wells when cells reached about 90–100% confluence. FBS‐free DMEM medium and mitomycin C (5 µg/mL) were used to eliminate the influence of proliferation. Wound healing was then observed and photographed at indicated time points by an Industrial Digital Camera (SOPTOP, Beijing, China). Three trials were performed for each condition. The remaining scratch area was calculated through ImageJ software (NIH, USA).

### Transwell Invasion Assay

Transwell invasion assay was performed using 8.0 µm Transwell Permeable Supports (Corning, NY, USA). Basement Membrane Matrigel LDEV Free (LABLEAD, Beijing, China) was diluted by precooled FBS‐free DMEM medium with volume ratio of 1:10, and 100 µL diluted Matrigel was added to each chamber and incubated at 37 °C for 2 h. About 2.5 × 10^4^ untreated or treated hKFs were seeded into the upper chamber, and the lower chamber was supplemented with 600 µL DMEM containing 10% fetal bovine serum. The invaded hKFs were then fixed by 4% paraformaldehyde and stained with Crystal Violet Staining Solution (Beyotime, Shanghai, China). The images were obtained by an Industrial Digital Camera (SOPTOP, Beijing, China) after 14–16 h incubation of 5% CO_2_ at 37 °C.

### Apoptosis Analysis

Apoptosis analysis was carried through an Annexin V‐Cyanine 5/PI Apoptosis Kit (Procell, Wuhan, China) according to the recommended protocol. Briefly, 5 × 10^5^ hKFs were collected and washed with PBS, centrifuged at 300× g for 5 min, and the supernatant was discarded. Cells were resuspended in 500 µL of diluted 1 × Annexin V Binding Buffer. About 5 µL of Annexin V‐ Cyanine 5 staining solution and 5 µL propidium iodide (PI) solution were added to the cell suspension, and the mixture was then incubated at room temperature in the dark for 20 min and analyzed through flow cytometry analysis. The CytoFLEX LX Flow Cytometer (BECKMAN COULTER, CA, USA) was used for apoptosis flow cytometry analysis. The results were then analyzed by FlowJo v10.8.1 (BD Biosciences, NY, USA).

### Immunofluorescence (IF) Analysis

Immunofluorescence analyses were performed as described previously.^[^ ^75^ ^]^ Briefly, hKFs grown on sterilized glass coverslips were washed with cold PBS and fixed with 4% paraformaldehyde. Cells were then permeabilized with 0.5% Triton X‐100 for 10 min. After blocking with 5% BSA (Amersco, Solon, OH, USA) for 1 h, cells were probed with the indicated antibodies overnight at 4 °C. After being washed with PBST (PBS containing 0.5% Tween‐20), secondary antibodies with specific fluorescence dyes were added and incubated for 1 h at room temperature. DAPI (Beyotime, Shanghai, China) were used to determine the location of the nucleus. The images were taken by an Industrial Digital Camera (SOPTOP, Beijing, China).

### Cell Cycle Analysis

The cell cycle analysis was performed by using a Cell Cycle and Apoptosis Analysis Kit (Beyotime, Shanghai, China), and analyzed by flow cytometry. Briefly, hKFs were collected and fixed with 70% ice ethanol overnight at 4 °C. The fixed cells were subsequently stained with PI, and incubated at 37 °C in the dark for 30 min. The DNA content was measured to determine the percentage of cells in the G0/G1, S, and G2/M phases. The CytoFLEX LX Flow Cytometer (BECKMAN COULTER, CA, USA) was used for cell cycle flow cytometry analysis. The results were analyzed by FlowJo v10.8.1 (BD Biosciences, NY, USA).

### EdU Cell Proliferation Assay

Fluorescence photography of EdU incorporation was assessed using a Cell‐Light EdU Apollo488 In Vitro Kit (RIBOBIO, Guangzhou, China) according to the manufacturer's protocol. The Apollo staining could cause inactivation of GFP, so the EdU signal would not be disturbed by GFP fluorescence signal. Briefly, hKFs were seeded and cultured overnight. EdU was added to the culture medium at a final concentration of 10 µM, and hKFs were incubated for 2 h at 37 °C. Following incubation, cells were fixed with 4% paraformaldehyde for 15 min at room temperature and permeabilized with 0.5% Triton X‐100 for 20 min. The cells were then subjected to the click reaction for 30 min at room temperature in the dark using 1 × Apollo 488 staining as the fluorophore. Nuclei were counterstained with Hoechst 33 342 for 10 min. The fluorescence images were captured using an Industrial Digital Camera (SOPTOP, Beijing, China), and EdU‐positive cells were quantified using ImageJ software. The percentage of EdU‐positive cells was calculated and used as a measure of cell proliferation.

Flow cytometry of EdU assay was performed by using a BeyoClick EdU Cell Proliferation Kit with Alexa Fluor 647 (Beyotime, Shanghai, China) according to the manufacturer's protocol. The experiment protocol was similar to the fluorescence photography of EdU assay. And EdU‐positive cells were quantified using a CytoFLEX LX Flow Cytometer (BECKMAN COULTER, CA, USA). The results were analyzed by FlowJo v10.8.1 (BD Biosciences, NY, USA).

### Immunoprecipitation (IP) Assay

The hKFs were lysed in Cell lysis buffer for Western and IP (LABLEAD, Beijing, China). The lysates were cleared by centrifugation at 14 000 rpm for 15 min at 4 °C. Supernatants were incubated overnight with BeyoMag Anti‐Flag Magnetic Beads (Beyotime, Shanghai, China) at 4 °C with constant rotation. After incubation, the beads were washed five times with washing buffer. The bound proteins were eluted by 3 × Flag Peptide (Beyotime, Shanghai, China), and then boiling the elution in SDS sample buffer for 10 min. Proteins were separated by SDS‐PAGE gel and followed by Western blotting with anti‐Flag antibody (TransGen, Beijing, China).

### Western Blot Analysis and Dot Blot Assay

Total proteins were extracted from hKFs using RIPA buffer (Beyotime, Shanghai, China) supplemented with cocktail protease inhibitors (Solarbio, Beijing, China), phosphatase inhibitors (Solarbio, Beijing, China), and PMSF (Solarbio, Beijing, China). The protein concentration was measured using the BCA Protein Assay Kit (LABLEAD, Beijing, China).

For western blot analysis, equal amounts of protein were separated by 15% SDS‐PAGE and transferred onto nitrocellulose (NC) membranes, 0.2 µm (Bio‐Rad, CA, USA). After blocking with no‐protein blocking solution (Beyotime, Shanghai, China) for 1 h at room temperature, the membranes were incubated with primary antibodies at 4 °C overnight. The membranes were then incubated with corresponding horseradish peroxidase (HRP)‐conjugated secondary antibodies for 1 h at room temperature. The detailed information on antibodies is shown in Table S4 (Supporting Information). Protein bands were detected using Clarity Western ECL Substrate Bio‐Rad, CA, USA) and visualized with the Amersham ImageQuant 800 (Cytiva, MA, USA).

For dot blot assay, 3 µg of total protein of each sample was directly spotted onto a NC membrane, 0.2 µm (Bio‐Rad, CA, USA), and air‐dry for 15 min at room temperature. The membrane was then blocked, incubated with primary antibody and corresponding HRP‐conjugated secondary antibody, and detected by ECL in the same way as a western blot.

### In Vitro Transcription (IVT) Assay

The linearized hsa_circ_00 26782 RNA sequence (transcribed from pLCDH‐26782 overexpressing plasmid) and corresponding antisense RNA sequence (transcribed from complementary chain) were transcribed in vitro using the Biotin RNA Labeling Kit (SP6/T7) (Beyotime, Shanghai, China), following the manufacturer's instructions to get biotinylated IVT RNAs. The RNAs were purified using the RNAeasy Animal RNA Isolation Kit with Spin Column (Beyotime, Shanghai, China), and were quantified using a NanoDrop 2000 spectrophotometer (ThermoScientific, MA, USA). The biotinylated IVT RNAs were stored at −80 °C until use.

### RNA Pulldown Assay and RNA‐RNA Pulldown Assay

According to the length/mass ratio of 1 µg/1000 nt, the purified linearized biotinylated hsa_circ_00 26782 RNA sequence and antisense RNA sequence were denaturalized and transformed into RNA secondary structure in RNA structure buffer. Both biotinylated RNAs were incubated with Streptavidin magnetic beads with rotation at 25 °C for 30 min to form bead‐bound RNA probes.

For RNA pulldown assay, RNA pulldown assay was performed by using the RNA pulldown kit (BersinBio, Guangzhou, China) following the users’ guide. Briefly, total protein samples were extracted from hKFs by using RIP buffer containing protease inhibitor. The remaining nucleic acids and chromatin were removed using DNase and agarose beads. The protein samples were then incubated with the indicated beads‐bound RNA probes at 25 °C for 2 h with gentle rotation. Following incubation, the beads were washed four times with 1 mL of ice‐cold NT2 buffer to remove non‐specifically bound proteins. Bound proteins were eluted by adding protein elution buffer, and the mixture was incubated at 37 °C for 2 h with gentle rotation. Each eluted protein samples were divided into two parts, one for subsequent mass spectrometry analysis, and the other was boiled in an equivalent 2 × SDS sample buffer for western blot analysis.

For RNA‐RNA pulldown assay, total RNA was extracted from hKFs using TRIzol method. About 1000 µg of total RNA was incubated with the indicated beads coated with 200 pmol purified linearized biotinylated hsa_circ_00 26782 RNA probe at 4 °C overnight with gentle rotation. The pulled‐down RNA was separated from the beads by TRIzol at 4 °C overnight with gentle rotation, then extracted RNA again using TRIzol method. The enrichment of target RNA was assessed by RT‐qPCR.

### Mass Spectrometry Analysis

Eluted protein samples from RNA pulldown assay were analyzed by Qinglian Biotech Co., Ltd (Beijing, China). Briefly, the mass spectrometry was performed through liquid chromatograpgy‐tandem mass spectrometry by using RIGOL L‐3000 HPLC system (RIGOL, Beijing, China). And the differential analysis between the sense RNA group and antisense RNA group were followed. The differential proteins were mapped to Homo_sapiens_SP_uniprot database (https://www.uniprot.org) by using Proteome Discoverer 2.4 software (ThermoScientific, MA, USA). GO and KEGG enrichment analyses were then applied to differential proteins.

### RNA Binding Protein Immunoprecipitation (RIP) Assay and Methylated RNA Immunoprecipitation (MeRIP) Assay

The RNA Binding Protein Immunoprecipitation (RIP) Kit (GEAN CREAT, Wuhan, China) was used to perform RIP assay to investigate the interaction between hsa_circ_00 26782 and candidate proteins. Briefly, hKFs were lysed by RIP Lysis Buffer containing Protease Inhibitor and RNase Inhibitor on ice for 30 min. Each lysis was divided into three parts, one for input group, one for IgG group, and the other for IP group. The ProteinA/G Magnetic Beads were incubated with equal amount of the indicated primary antibodies or IgG on a silent mixer and incubated for 2 h at room temperature. The divided lysis was then added to the coated ProteinA/G Magnetic Beads, and incubated on a silent mixer at 4 °C overnight. The beads were then washed by RIP Wash Buffer for three times, and the protein‐RNA complexes were eluted by Elution Buffer for further western blot analysis or RT‐qPCR analysis.

The Methylated RNA immunoprecipitation (MeRIP) Kit (BersinBio, Guangzhou, China) was used to perform MeRIP assay to investigate the m^6^A modifications of hsa_circ_00 26782 following the users’ guide, which was similar to RIP assay.

### RNA Higher Structure and RNA‐Protein Interaction Conformation Change Prediction

The linear secondary structure was predicted using the RNAfold web server (http://rna.tbi.univie.ac.at/cgi‐bin/RNAWebSuite/RNAfold.cgi) ^[^ ^76^, ^77^ ^]^ by inputting the linearized RNA sequence. The tertiary structure was predicted using the 3dRNA web server (http://biophy.hust.edu.cn/3dRNA) ^[^ ^78^, ^79^ ^]^ by inputting the linearized RNA sequence and corresponding secondary structure, and visualized by PyMOL (Schrödinger, LLC, NY, USA). The crystal structure of the CREB1 homodimer (PDB ID: 5ZKO) was obtained from the RCSB PDB web server (https://www.rcsb.org/).^[^ ^80^ ^]^

The molecular docking simulations were conducted using the HDOCK server (http://hdock.phys.hust.edu.cn/), 100 conformations were generated. These conformations were ranked based on docking scores, and the top 10 were selected. The optimal conformation was chosen based on the binding site, and interaction diagrams were generated using LigPlus software (University College London, London, UK), the binding illustrations were created using PyMOL software. The molecular dynamics (MD) simulation focused on analyzing the binding affinity between a protein‐small molecule complex. After performing the necessary pre‐simulation preparations for both the complex and the apo protein, Gromacs 2021 (KTH Royal Institute of Technology, Stockholm, Sweden) was selected as the MD simulation software to form the Amber99sb‐ildn force field (applied to model the protein and small molecule interactions). The SPCE water model was used to solvate the system. A water box of 10 × 10 × 10 nm^3^ with at least a 1.2 nm buffer between the protein and the edges of the box was created, and ions were added to neutralize the system. Electrostatic interactions were treated using the Particle‐Mesh Ewald (PME) method, and energy minimization was conducted using the steepest descent method with a maximum of 50 000 steps. Both the Coulomb and van der Waals cutoff distances were set to 1 nm. The system was then equilibrated using the constant Number of particles, Volume, and Temperature (NVT) and constant Number of particles, Pressure, and Temperature (NPT) ensembles before running a 100 ns MD simulation at constant temperature and pressure. Non‐bonded interaction cutoff values were set to 10 Å. Temperature was maintained at 300 K using the V‐rescale coupling method, and pressure was controlled at 1 bar using the Berendsen method. Binding free energy was calculated using Gmx_MMPBSA (https://valdes‐tresanco‐ms.github.io/gmx_MMPBSA/dev/), with larger absolute values indicating stronger binding affinity between the RNA and the protein.

### Native Western Blot

The hKFs were lysed by Nondenature Lysis Buffer (Sangon, Shanghai, China), and total protein samples containing 5 × Native Sample Loading Buffer (Sangon, Shanghai, China) were boiled on metal bath at 100 °C for 10 min. The native western blot was performed by using the Native PAGE Preparation kit (Sangon, Shanghai, China), the SDS‐PAGE was performed in 10 × Tris‐Glycine Native PAGE Running Buffer PH 8.8 (Sangon, Shanghai, China), and the other protocols were same as western blot.

### Nucleocytoplasmic Separation

Nuclear and cytosolic fractions were isolated from the hKFs using a PARIS Kit (ThermoScientific, MA, USA) according to the manufacturers’ instruction. Briefly, the hKFs were lysed with Cell Fractionation Buffer, and the lysate was divided into two parts, one for the nucleus and cytoplasmic RNA extraction, and the other for the nucleus and cytoplasmic protein extraction. After incubated on ice for 10 min, the samples were centrifuged at 4 °C for 5 min. The supernatant containing the cytoplasmic fraction were carefully aspirated away from the nuclear pellet. The nuclear pellet was lysed in Cell Disruption Buffer, vortexed to lyse the nuclei until the lysate was homogeneous. The cytoplasmic lysate and nucleic lysate containing proteins could be used in further protein analysis. For RNA isolation, both cytoplasmic lysate and nucleic lysate were mixed with an equal volume of 2 × Lysis/Binding Solution, followed by one “sample volume” of 100% ethanol. The mixtures were then applied to Filter Cartridge assembled in Collection Tube and eluted with preheated Elution Solution to get RNA samples.

### Chromatin Bound Protein Extraction

Chromatin bound proteins were extracted using the Chromatin Extraction Kit (Abcam, Cambridge, UK) according to the users’ guide. Briefly, hKFs to 80–90% confluence were lysed by Working Lysis Buffer containing Protease Inhibitor Cocktail on ice for 10 min. Vortex vigorously for 10 s and centrifuge at 5000 rpm for 5 min. The Working Extraction Buffer was then added to chromatin pellet and the pellet was resuspended by carefully pipetting up and down. After incubating the sample on ice for 10 min and sonicating the sample for 2 × 20 s to increase chromatin extraction, the sample was centrifuged at 12 000 rpm at 4 °C for 10 min, and the supernatant containing chromatin was added to Chromatin Buffer at a 1:1 ratio. The chromatin sample was stored at −80 °C until use.

### Construction of CREB1 Overexpression Plasmid with a Serine Residues 142 Mutation

The CREB1 overexpression plasmid with a serine residues 142 mutation was constructed following previous studies.^[^ ^27^, ^55^ ^]^ Briefly, the DNA sequence coding serine residues (TCT) was edited to GCT, which codes for alanine. The mutant CREB1 overexpression plasmids were chemically synthesized by Tsingke Biotech Co., Ltd.

### Subcutaneous Xenograft Keloid Models

Female BALB/c nude mice, aged 6 weeks, and weighing 15–19 g, were obtained from the Laboratory Animal Center of Zhejiang University, for in vivo subcutaneous xenograft experiments. The animals were treated in accordance with relevant institutional and national guidelines and regulations. The animal experiments were approved by the Ethics Committee for Animal Use of Laboratory Animal Center, Zhejiang University (No. 30 715). As described previously,^[^ ^81^ ^]^ 1 × 10^7^ hKFs were taken in the logarithmic growth phase, and were then resuspended in 100 µL suspension of Matrigel High Concentration Phenol‐Red Free, LDEV‐free (LABLEAD, Beijing, China) and complete DMEM medium (volume ratio:1:1). Each nude mouse was injected with three groups of hKFs subcutaneously, untreated hKFs (NC) were injected at infra‐axillary subcutaneous tissue, hKFs with knock‐out (KO) or over‐expression (OE) of hsa_circ_00 26782 were injected at right or left groin subcutaneous tissue, respectively. The nude mice were executed by overdose anesthesia, and the xenograft tumors were excised for staining. The long axis (a) and short axis (b) of keloids were measured and the volume (V = a × b^2^/2) was calculated.

### Hematoxylin and Eosin (H&E) Staining and Masson's Trichrome Staining

Xenograft tumors excised from the nude mice were fixed in 4% paraformaldehyde, embedded in paraffin, and cut into 4 µm thick sections. The sections were deparaffinized in xylene, rehydrated through graded ethanol series, and then stained with hematoxylin for 5 min. After rinsing in running water, sections were differentiated in 1% acid alcohol, followed by bluing in lithium carbonate solution. The sections were then counterstained with eosin for 2 min, dehydrated through graded ethanol, cleared in xylene, and mounted with coverslips using a synthetic mounting medium. Stained sections were scanned using a VS120 High‐speed slice scanning system (Olympus Corporation, Tokyo, Japan).

Masson's trichrome staining was performed by using the Masson's Trichrome Stain Kit (Solarbio, Beijing, China). Briefly, paraffin‐embedded tissue sections (4 µm thick) were deparaffinized in xylene and rehydrated through graded ethanol series. The sections were then stained with Weigert's iron hematoxylin solution to visualize nuclei. After rinsing in running water, sections were stained with Biebrich scarlet‐acid fuchsin solution. The sections were differentiated in phosphomolybdic‐phosphotungstic acid solution, followed by staining with aniline blue to visualize collagen fibers. After staining, sections were rinsed briefly in 1% acetic acid, dehydrated through graded ethanol, cleared in xylene, and mounted with a synthetic resin. Stained sections were scanned using a VS120 High‐speed slice scanning system (Olympus Corporation, Tokyo, Japan).

### Ki67 Immunohistochemistry Staining and TUNEL Assay

The preparation of paraffin‐embedded xenograft tumor sections was performed as previously described. Ki67 immunohistochemical staining and TUNEL DAB staining of the paraffin sections were conducted by the Department of Pathology, Sir Run Run Shaw Hospital, Zhejiang University School of Medicine. Stained sections were scanned using a VS120 high‐speed slide scanning system (Olympus Corporation, Tokyo, Japan).

### Dual‐Luciferase Reporter Assay

The dual‐luciferase reporter assay was performed using the Dual Luciferase Reporter Gene Assay Kit (Beyotime, Shanghai, China) according to the manufacturer's instructions. Briefly, indicated hKFs were seeded at 5 × 10^4^ cells per well in 12‐well plates and allowed to settle overnight. Then hKFs were co‐transfected with 1 µg of the pCREB‐TA‐Luc plasmids (containing five CRE elements and firefly luciferase reporter gene) and 1 µg of the pRL‐SV40‐N plasmids (containing renilla luciferase reporter gene) using Lipo8000. After 24 h of transfection, the cells were lysed with Reporter Gene Lysis Buffer. The luciferase activity was measured using a SynergyMx M5 (Molecular Devices, Fremont, CA, USA). Firefly luciferase activity was normalized to Renilla luciferase activity to account for variations in transfection efficiency.

The pmirGLO plasmids containing wild‐type or mutant promoter/enhancer sequence of *DUSP9*, *PHLDA1*, and *LDLR* were constructed by Azenta Life Sciences. And the dual‐luciferase reporter assay was performed as previously described.

### Whole‐Transcriptome Sequencing

The whole‐transcriptome sequencing (including RNA extraction, cDNA library construction, sequencing, and data analysis) was performed by Personalbio (Shanghai, China). The sequencing procedures were similar to circRNA high‐throughput sequencing, and are therefore not described in detail here.

### Statistical Analysis

In this study, the data were presented as the mean ± standard deviation from three independent experiments. All statistical analysis was performed using GraphPad Prism 9 software (https://www.graphpad.com/scientific‐software/prism/). The authors adopted rank sum test for DEcirc expression level comparisons between keloid and normal scar tissues, t‐test, or One‐Way ANOVA for multiple comparisons. A *p*‐value < 0.05 was considered statistically significant.

## Conflict of Interest

The authors declare no conflict of interest.

## Author Contributions

X.Z., C.C., and Z.F. These authors contributed equally to this work. X.C.Z. wrote the manuscript, performed the most experiments, and analyzed the data; C.Y.C. performed the Masson's staining, the EdU assay, and Z.X.F. performed the wound scratch assay and CCK‐8 assay; Y.W. performed transient transduction and stable cell line establishment; T.Z. constructed the expression vectors and CRISPR/Cas9 recombinant plasmids; Z.M.Z. performed the HE staining; Z.R.W. performed the tumor‐bearing assay; Y.Z.D. provided experimental design guidance; J.C. provided experimental design guidance; X.Y.L. and W.Q.T. provided the experimental platform, instruments, reagents, and article writing guidance.

## Supporting information



Supporting Information

## Data Availability

The data that support the findings of this study are available from the corresponding author upon reasonable request.;
