# Supplementary material for: Hsa_circ_0026782 Acts as a “Molecular Break” of CREB1‐Mediated Transcription by Promoting Its Phosphorylation at Ser142 That Prevents Keloid Progression
Source: Adv Sci (Weinh). 2025 Aug 18;12(40):e08647. doi: 10.1002/advs.202508647 (PMC12561425; doi:10.1002/advs.202508647)
Supplement: Supplementary file 1 — Supporting Information [file ADVS-12-e08647-s001.docx]

**Supporting Information for**

**Hsa_circ_0026782 acts as a “molecular break” of CREB1-mediated transcription by promoting its phosphorylation at Ser142 that prevent keloid progression**

**Xin-Cao Zhong *et al.***

***Corresponding author.**

**Email:** [**tanweixxxx@zju.edu.cn**](mailto:tanweixxxx@zju.edu.cn)**;** [**xiaoyinglin@zju.edu.cn**](mailto:xiaoyinglin@zju.edu.cn)**;** [**chenjun2009@zju.edu.cn**](mailto:chenjun2009@zju.edu.cn)

**This file includes:**

**Figure S1 to S9**

**Table S1 to S4**

**Supplementary Methods**

**References (1 to 12)**


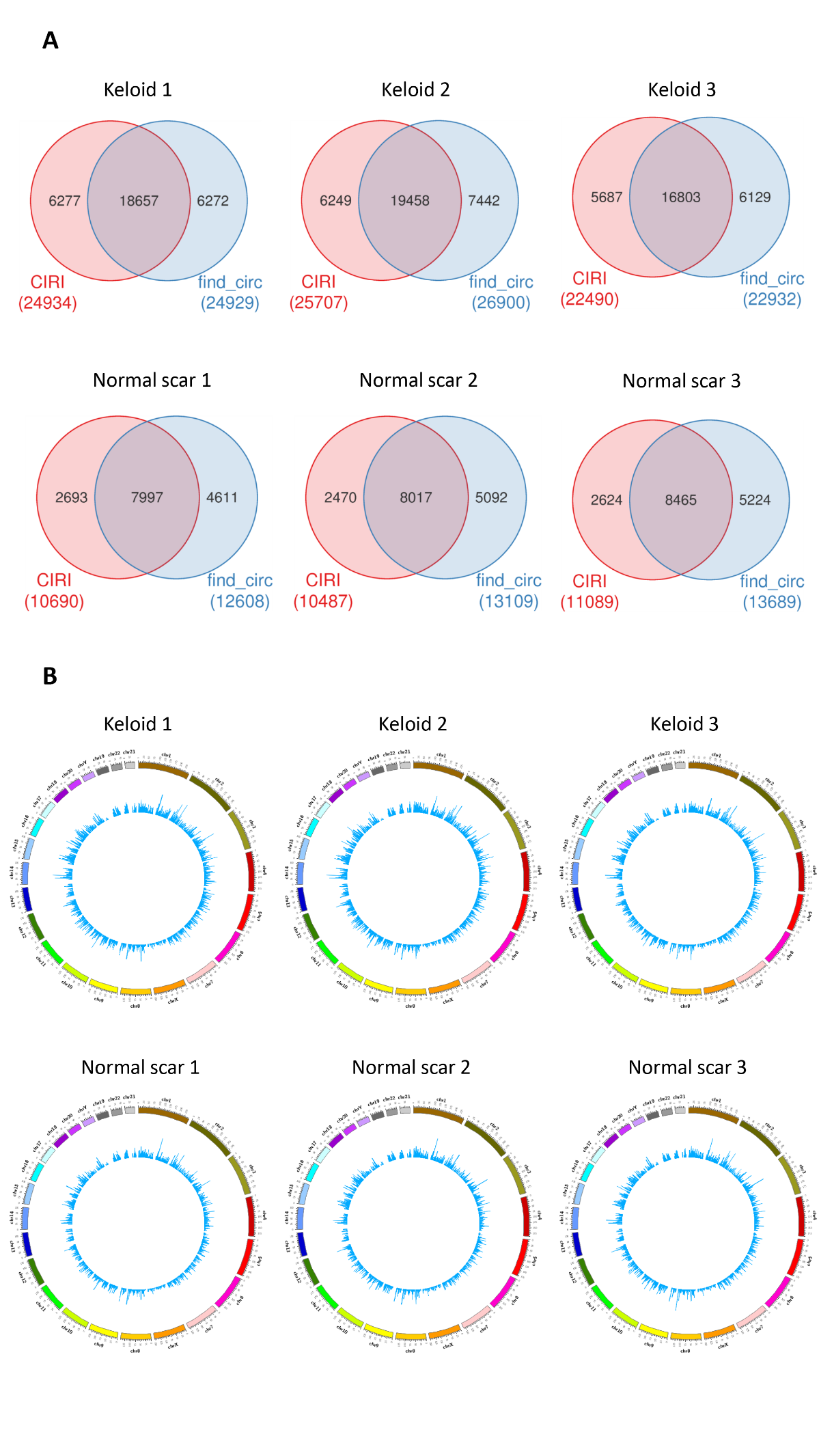


**Figure S1. Prediction and genome distribution of circRNAs.** (A) Venn diagrams of circRNAs predicted by CIRI (pink) and find_circ (blue), and the number of circRNAs was indicated below the software. (B) The distribution of circRNAs across the genome were shown via Circos. The outer circle represents the chromosome. The inner circle represents the change in the number of circRNAs on the chromosome with a sliding window of 1,000,000 bases.


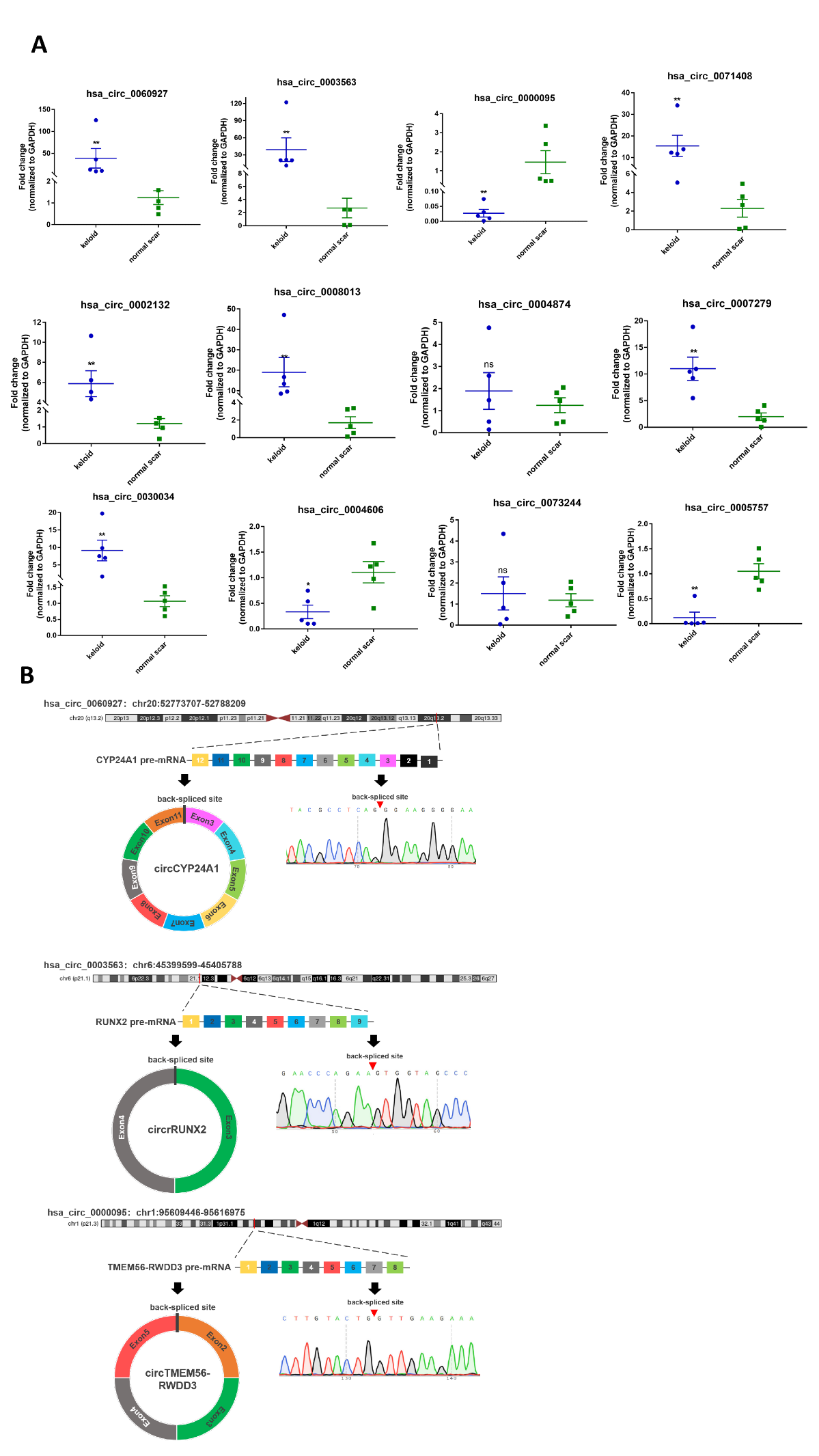


**Figure S2. Expression** **levels and details of DEcircs.** (A) The expression levels of twelve selected DEcircs (excluding hsa_circ_0026782) in five pairs of keloids (blue) and normal scars (green). (B) Genome location, components, and Sanger sequencing of hsa_circ_0060927, hsa_circ_0003563, and hsa_circ_0000095. The BSJ site was marked by a red inverted triangle.


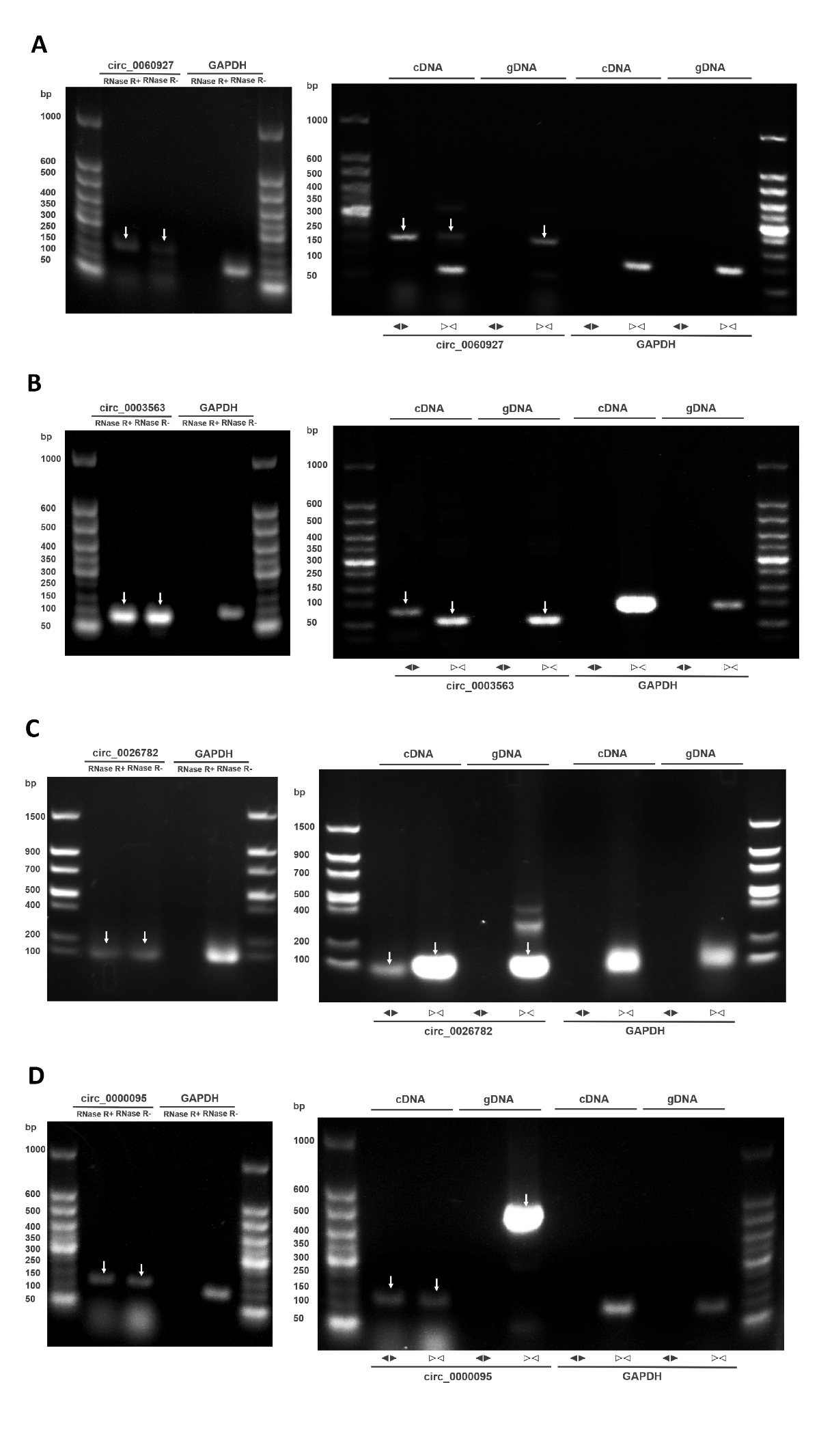


**Figure S3. Further Validation of DEcircs.** (A) Agarose gel electrophoresis of hsa_circ_0060927 RT-qPCR products on RNase R-digested or undigested RNAs (left). Agarose gel electrophoresis of hsa_circ_0060927 RT-qPCR products using divergent or convergent primers in cDNAs or gDNAs (right). (B) Agarose gel electrophoresis of hsa_circ_0003563 RT-qPCR products on RNase R-digested or undigested RNAs (left). Agarose gel electrophoresis of hsa_circ_0003563 RT-qPCR products using divergent or convergent primers in cDNAs or gDNAs (right). (C) Agarose gel electrophoresis of hsa_circ_0026782 RT-qPCR products on RNase R-digested or undigested RNAs (left). Agarose gel electrophoresis of hsa_circ_0026782 RT-qPCR products using divergent or convergent primers in cDNAs or gDNAs (right). (D) Agarose gel electrophoresis of hsa_circ_0000095 RT-qPCR products of RNase R-digested or undigested RNAs (left). Agarose gel electrophoresis of hsa_circ_0000095 RT-qPCR products generated from cDNA or gDNAs via divergent or convergent primers (right). The product bands were marked by white arrows. The back-to-back black triangles represented divergent primers, and the point-to-point white triangles represented convergent primers.


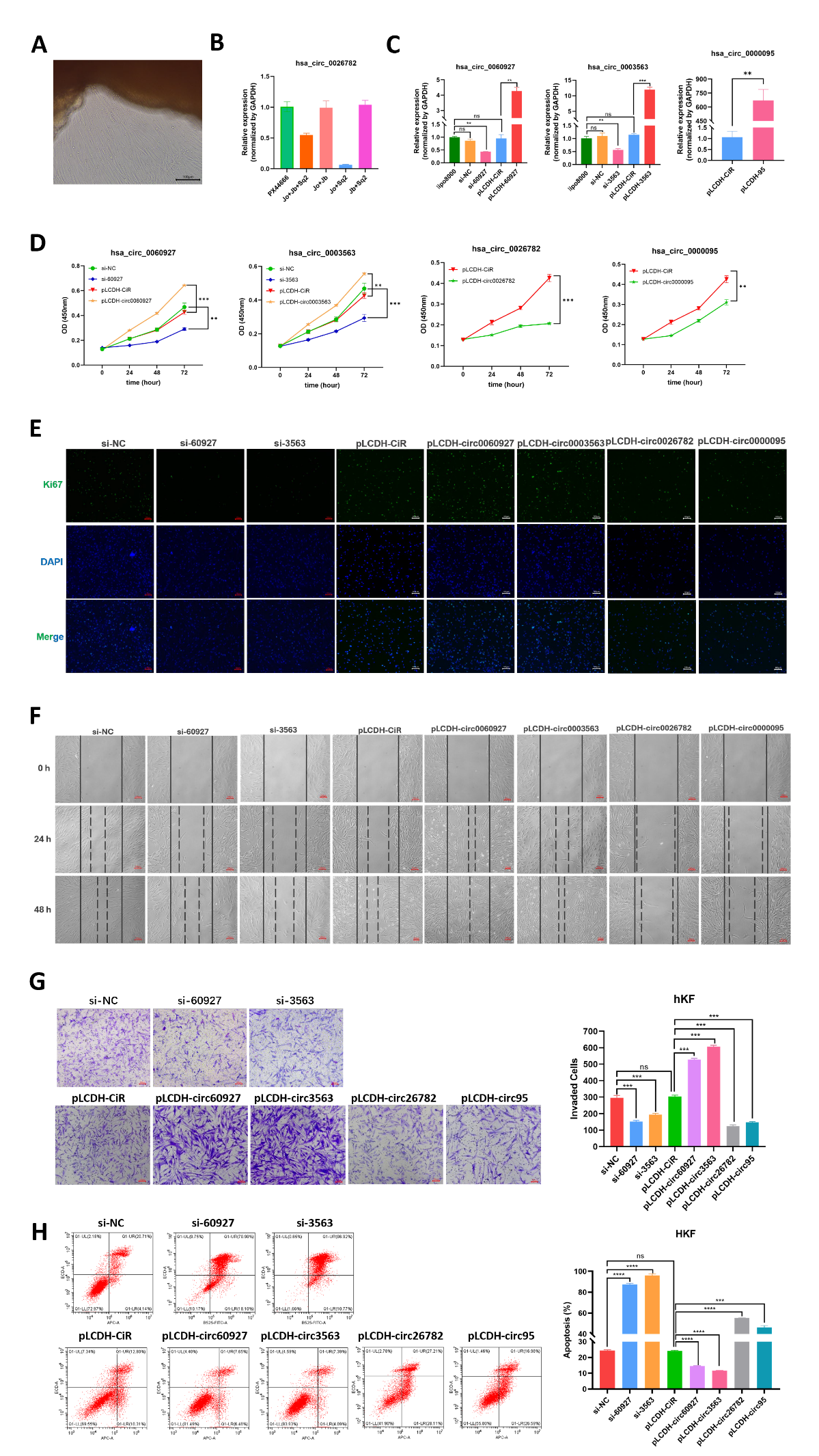


**Figure S4. Biological** **effects of DEcircs.** (A) Primary human keloid fibroblasts (hKFs) isolation. hKFs were spindle-shaped and presented the typical morphological features of fibroblasts. Original magnification: 40 ×. (B) The knockdown efficiency of hsa_circ_0026782 by the indicated combinations of sgRNAs targeting different Alu elements. Compared with the empty CRISPR/Cas9 vector pX44666, the maximal knockdown efficiency was achieved by knocking out AluJo and AluSq2 simultaneously. (C) Knockdown efficiency of siRNAs targeting hsa_circ_0060927 (si-60927) or hsa_circ_0003563 (si-3563) compared with the negative control (si-NC). The overexpression efficiency of hsa_circ_0060927 (pLCDH-60927), hsa_circ_0003563 (pLCDH-3563), and hsa_circ_0000095 (pLCDH-95) was compared with that of the empty overexpression vector pLCDH-CiR. (D) CCK-8 proliferation assay of the indicated hKFs. The absorbance at OD450 was detected at 24, 48 and 72 hours after cell seeding. (E) Fluorescence image of Ki67 immunofluorescence (green) was used to observe the effect of the indicated DEcircs on hKFs proliferation. Cell nuclei were stained with 4,6-diamidino-2-phenylindole (DAPI; blue). Original magnification: 200 ×. (F) Wound scratch assay of the indicated hKFs. The wound closure area was photographed at 0, 24, and 48 hours after cell seeding. (G) Transwell invasion assay of the indicated hKFs (left) and quantification of the invasive cell number (right). (H) Cell apoptosis flow cytometry analysis of the indicated hKFs (left) and the quantification of apoptosis ratio (right). Experiments were performed in triplicate. Error bars indicate the mean ± SD. ns *p* ＞ 0.05, ***p* < 0.01, ****p* <0.001, *****p* <0.0001 by independent t-tests.


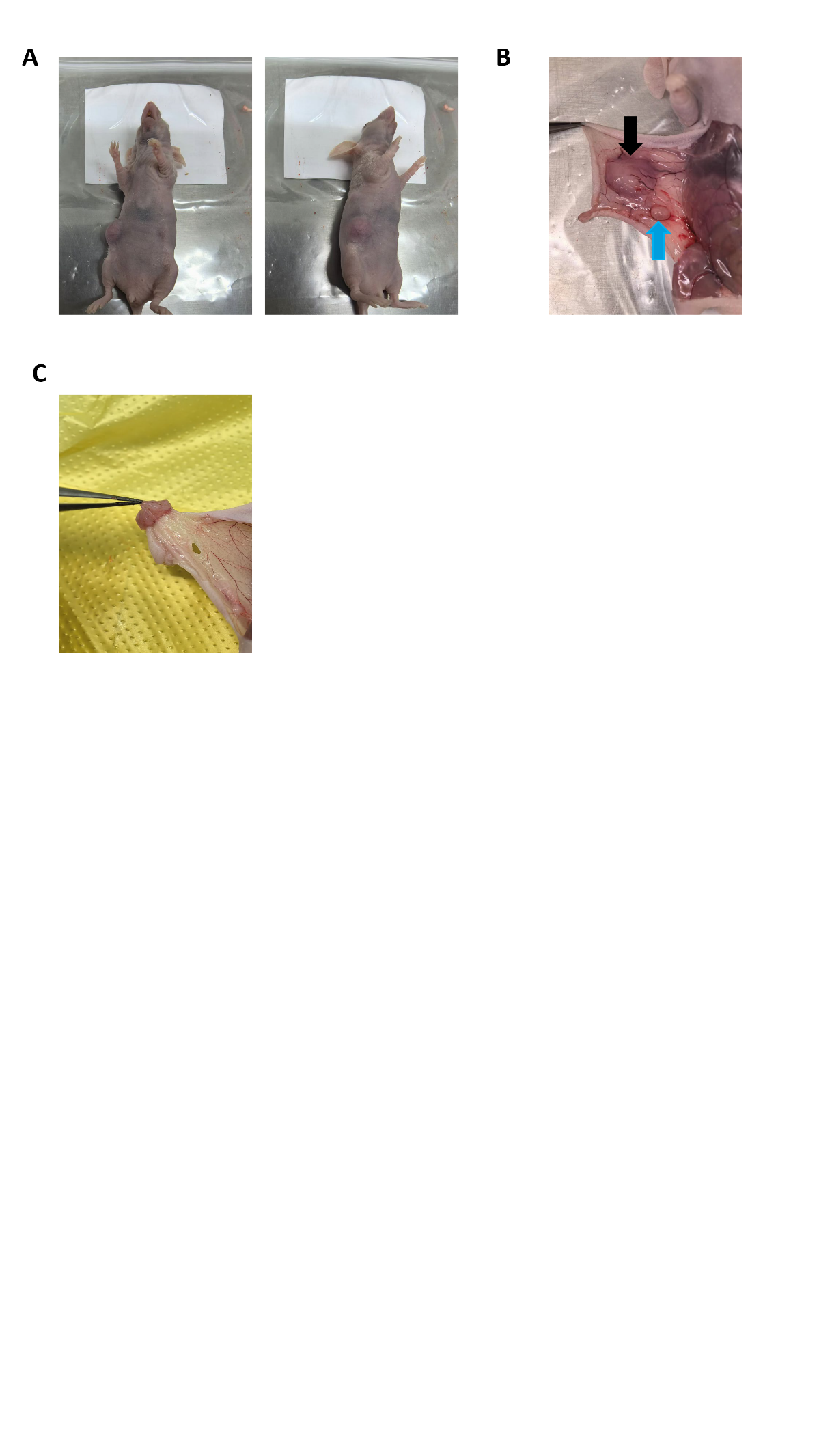


**Figure S5. Tumor-bearing experiments in nude mice.** (A) Six-week-old female BALB/c nude mice were subcutaneously injected with 1×10^7^ of the indicated hKFs. The sacrificed animals bearing xenografts at week eight were photographed with an iPhone 15before tumor excision. (B) The subcutaneous xenografts of sacrificed nude mice at week eight were indicated by black arrow, and the inguinal lymph node was indicated by blue arrow. (C) The excision of xenograft. The tumor clearly grew at the subcutaneous level. Images were captured with an iPhone 15.


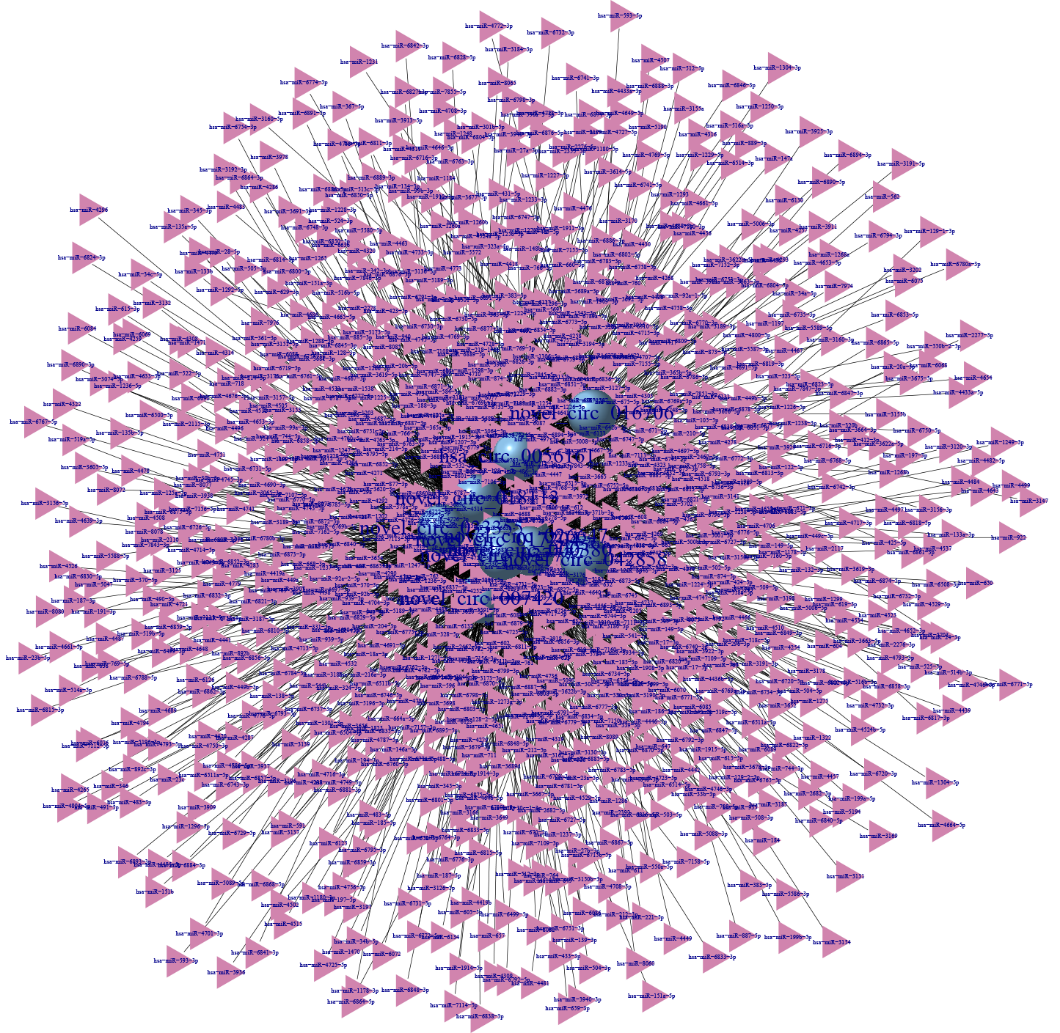


**Figure S6. CircRNA‒miRNA interaction network.** The blue spheres represented circRNAs, and the red triangles represented miRNAs. The volume of each blue sphere indicated the number of interacting miRNAs. The more miRNAs a circRNA interacted with, the larger the sphere.


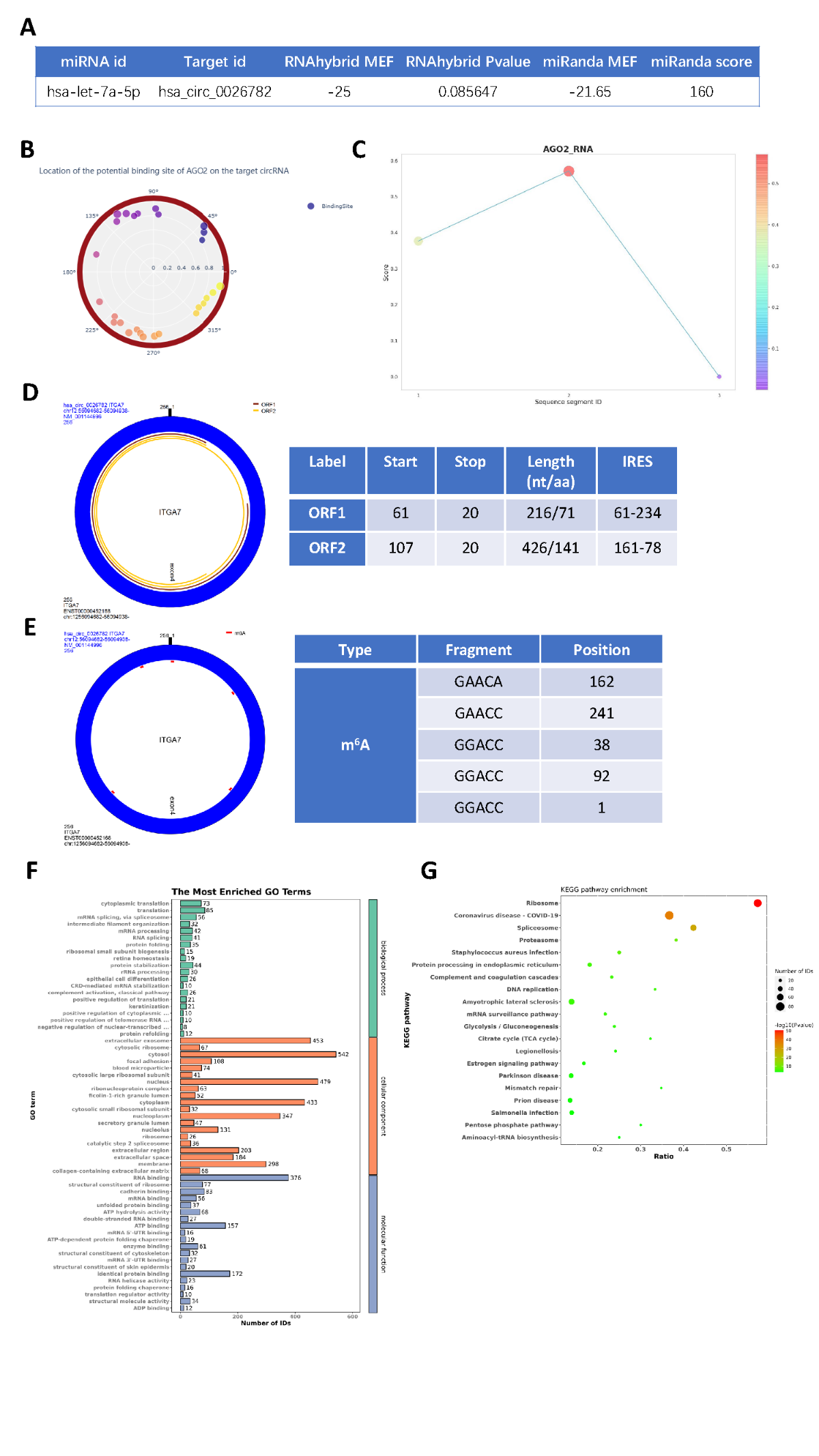


**Figure S7. Hsa_circ_0026782 may not code oligopeptides or act as a miRNA-sponge.** (A) Prediction of miRNA that might bind to hsa_circ_0026782 by using miRanda and RNAhybrid. Hsa-let-7a-5p may potentially bind to hsa_circ_0026782. (B) Prediction of AGO2 binding sites on hsa_circ_0026782 by CRWS. Each dot represented a binding site, and the color of the dots has no specific meaning. The outer red ring represented hsa_circ_0026782, while the inner multiple white rings indicate different binding probabilities. (C) Prediction of the binding probability between hsa_circ_0026782 and AGO2 using RBPsuite. The x-axis represented hsa_circ_0026782, and the y-axis showed the prediction score. A score greater than 0.5 indicates a high binding probability. (D) ORF prediction diagram of hsa_circ_0026782 by CircPrimer (left) and details of the predicted ORFs and IRES (right). (E) Prediction of m^6^A modifications of hsa_circ_0026782 by CircPrimer (left) and details of the predicted modified sites (right). (F) GO enrichment analysis of differentially expressed proteins. The Y-axis represented the GO functional items, and the X-axis represented the number of proteins pulled down by RNA pulldown assay in the corresponding GO term. (G) KEGG pathway enrichment analysis of differentially expressed proteins. The Y-axis represented the pathway entry, and the X-axis represented the ratio of proteins pulled down by RNA pulldown assay to the corresponding pathway entry. The dot size represented the number of proteins, and the color represented the *p* value.


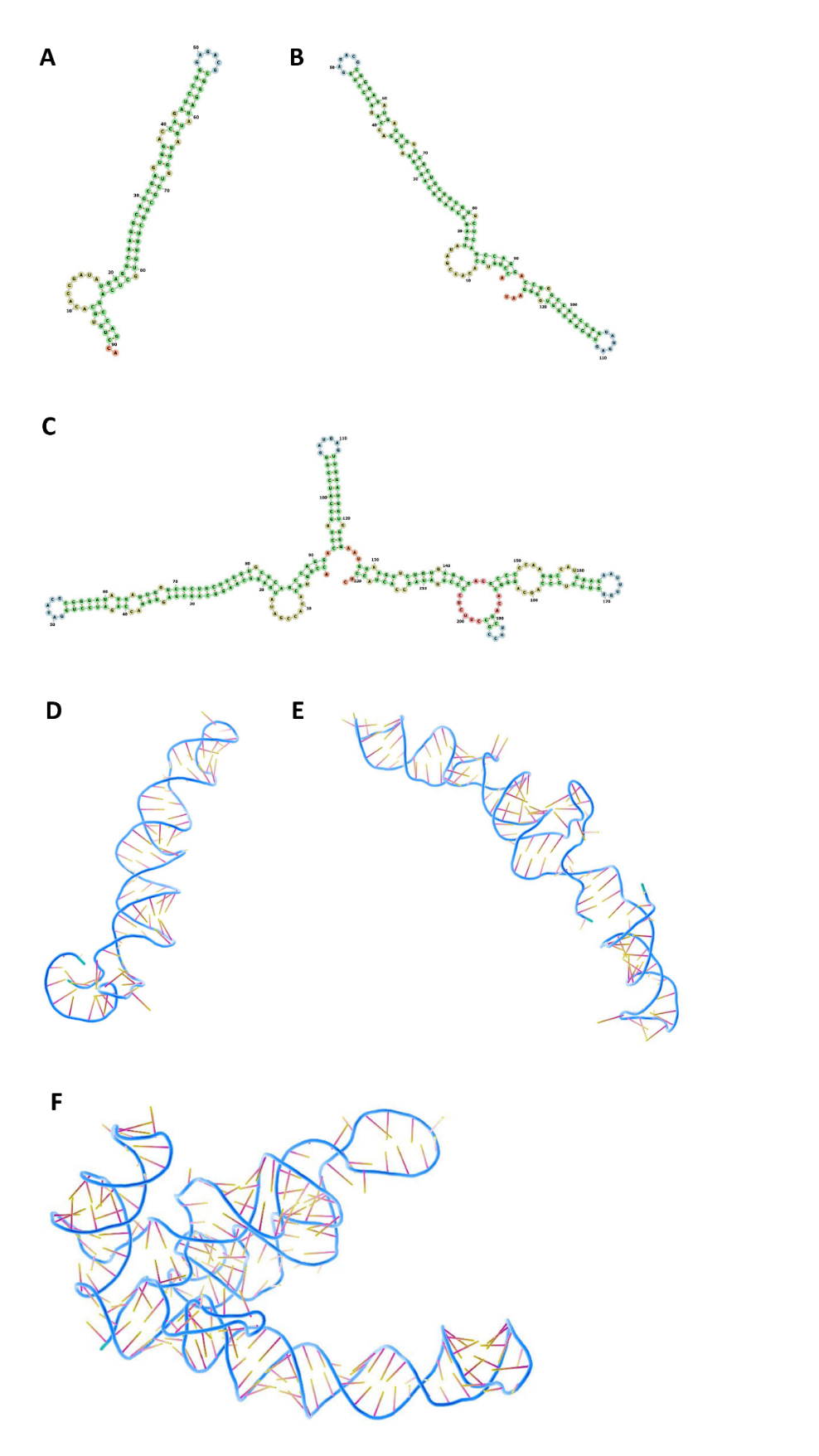


**Figure S8. Higher structure of hsa_circ_0026782 truncations.** (A) MFE plain secondary structure of 1-90 nt of linearized hsa_circ_0026782. (B) MFE plain secondary structure of 1-127 nt of linearized hsa_circ_0026782. (C) MFE plain secondary structure of 1-222 nt of linearized hsa_circ_0026782. (D) Tertiary structure of 1-90 nt of linearized hsa_circ_0026782. (E) Tertiary structure of 1-127 nt of linearized hsa_circ_0026782. (F) Tertiary structure of 1-222 nt of linearized hsa_circ_0026782.


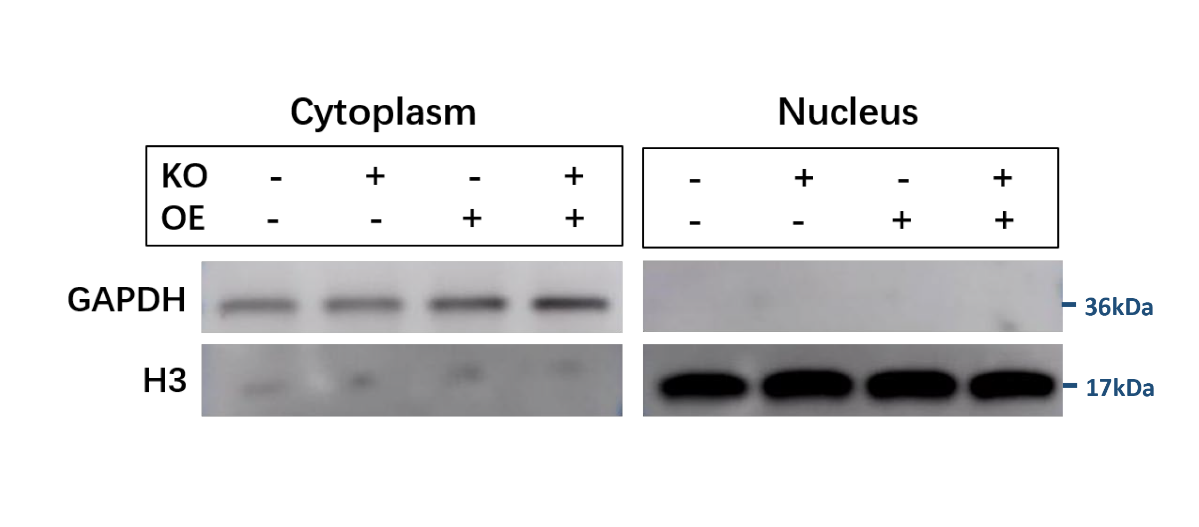
**Figure S9. Proteins extraction from the nucleus and cytoplasm.** The protein levels of GAPDH and H3 were used for quality control in the hKF fractionation experiment.

**Table S1 Characteristics of the patients and donors**

| **Number** | **Age** | **Gender** | **Body Part** | **Specimen** | **Experiments** |
| --- | --- | --- | --- | --- | --- |
| 1 | 36 | F | pinna | keloid | Fig 2-3, Fig 4A, Fig 5-6, Fig 7J-K, Fig 8-10, Sup Fig S2A, Sup Fig S3-S5, Sup Fig S9 |
| 2 | 20 | F | back | keloid | Fig 1, Sup Fig S1, Sup Fig S2A |
| 3 | 46 | F | chest | keloid | Fig 1, Sup Fig S1, Sup Fig S2A |
| 4 | 21 | F | pinna | keloid | Fig 2-3, Fig 4A, Fig 5-6, Fig 7J-K, Fig 8-10, Sup Fig S2A, Sup Fig S3-S5, Sup Fig S9 |
| 5 | 21 | M | chest | keloid | Fig 1, Sup Fig S1, Sup Fig S2A |
| 6 | 50 | F | chest | keloid | Fig 2-3, Fig 4A, Fig 5-6, Fig 7J-K, Fig 8-10, Sup Fig S2A, Sup Fig S3-S5, Sup Fig S9 |
| 7 | 42 | F | back | keloid | Fig 2-3, Fig 4A, Fig 5-6, Fig 7J-K, Fig 8-10, Sup Fig S2A, Sup Fig S3-S5, Sup Fig S9 |
| 8 | 45 | M | back | keloid | Fig 2-3, Fig 4A, Fig 5-6, Fig 7J-K, Fig 8-10, Sup Fig S2A, Sup Fig S3-S5, Sup Fig S9 |
| 9 | 28 | M | back | keloid | Fig 2-3, Fig 4A, Fig 5-6, Fig 7J-K, Fig 8-10, Sup Fig S3-S5, Sup Fig S9 |
| 10 | 37 | M | chest | keloid | Fig 2-3, Fig 4A, Fig 5-6, Fig 7J-K, Fig 8-10, Sup Fig S3-S5, Sup Fig S9 |
| 11 | 22 | F | chest | keloid | Fig 2-3, Fig 4A, Fig 5-6, Fig 7J-K, Fig 8-10, Sup Fig S3-S5, Sup Fig S9 |
| 12 | 20 | M | pinna | keloid | Fig 2-3, Fig 4A, Fig 5-6, Fig 7J-K, Fig 8-10, Sup Fig S3-S5, Sup Fig S9 |
| 13 | 25 | F | back | keloid | Fig 2-3, Fig 4A, Fig 5-6, Fig 7J-K, Fig 8-10, Sup Fig S3-S5, Sup Fig S9 |
| 14 | 34 | F | chest | keloid | Fig 2-3, Fig 4A, Fig 5-6, Fig 7J-K, Fig 8-10, Sup Fig S3-S5, Sup Fig S9 |
| 15 | 44 | M | pinna | keloid | Fig 2-3, Fig 4A, Fig 5-6, Fig 7J-K, Fig 8-10, Sup Fig S3-S5, Sup Fig S9 |
| 16 | 36 | M | back | normal scar | Fig 1, Sup Fig S1, Sup Fig S2A |
| 17 | 35 | M | back | normal scar | Fig 1, Sup Fig S1, Sup Fig S2A |
| 18 | 33 | M | back | normal scar | Fig 1, Sup Fig S1, Sup Fig S2A |
| 19 | 40 | M | belly | normal scar | Fig 1, Sup Fig S1, Sup Fig S2A |
| 20 | 38 | F | left upper arm | normal scar | Fig 1, Sup Fig S1, Sup Fig S2A |
| 21 | 32 | F | clavicle | normal scar | Fig 1, Sup Fig S1, Sup Fig S2A |
| 22 | 37 | M | right crus | normal scar | Fig 1, Sup Fig S1, Sup Fig S2A |
| 23 | 40 | M | right shoulder | normal scar | Fig 1, Sup Fig S1, Sup Fig S2A |
| 24 | 39 | M | belly | normal scar | Fig 1, Sup Fig S1, Sup Fig S2A |

* “F” represents female, “M” represents male.

* All results were confirmed by at least three independent experiments.

**Table S2 Primers used in RT- qPCR**

| **Target Gene** | **Direction** | **Primer Type** | **Sequence** |
| --- | --- | --- | --- |
| hsa_circ_0060927 | Forward | Divergent (back-to-back) | GGCCACAGACAATGAGCC |
|  | Reverse |  | AAATCGGCCAAGACCTCA |
| hsa_circ_0071408 | Forward | Divergent (back-to-back) | CCAAAACTAGGAGATTTGCACCC |
|  | Reverse |  | CAGTGCTGGTTGCTGATCCA |
| hsa_circ_002132 | Forward | Divergent (sjod) | ATCTGGCTCCGCATCCC |
|  | Reverse |  | GCCGCTGCAACTCGTCT |
| hsa_circ_0008013 | Forward | Divergent (sjod) | GTGAAGAAACCTCAGGAAAGCA |
|  | Reverse |  | TGGCAGTTCTCCTCTCCAAG |
| hsa_circ_0003563 | Forward | Divergent (sjod) | ACCGTCTTCACAAATCCTCC |
|  | Reverse |  | TGACATAACCACAGTCCCATC |
| hsa_circ_0004874 | Forward | Divergent (sjod) | TGAAGCAACTAAGAGTCCTGTG |
|  | Reverse |  | TTGTCCCTCATCTCTGCTGA |
| hsa_circ_0007279 | Forward | Divergent (sjod) | CAGCCTCAACTGAAGGAGCAT |
|  | Reverse |  | CTGGTTTCACACCGAAGGACT |
| hsa_circ_0030034 | Forward | Divergent (sjod) | AGTGAAGTTCGAATTGAGCAAGT |
|  | Reverse |  | TGGGAGTTCAACCTTGTCCA |
| hsa_circ_0026782 | Forward | Divergent (sjod) | AGGCCATGAACAATTTGGGT |
|  | Reverse |  | GGTGTGCACAGGTCCTTCC |
| hsa_circ_0000095 | Forward | Divergent (back-to-back) | TTGGGGTGGTCCATCACTTG |
|  | Reverse |  | TTCTTCAACCAGTACAAGGTAGT |
| hsa_circ_0005757 | Forward | Divergent (sjod) | AGGCTGTGAAGAGAACTGTTT |
|  | Reverse |  | GTTCCCCATGATAATTTCTCCCA |
| hsa_circ_0004604 | Forward | Divergent (sjod) | GGTGCTGCTGGTGGTGGA |
|  | Reverse |  | TGTCTGTGCGGATGAGGC |
| hsa_circ_0073244 | Forward | Divergent (sjod) | AAGACTATCAGATCACTGCCTCCAG |
|  | Reverse |  | CATTTTCCTTTGCAAATTTATCTGT |
| ITGA7 exon4 | Forward | Convergent | ACAAGGGTGCTGTGGTCATCC |
|  | Reverse |  | AGCCAGTGAGTAGCCAAAGCC |
| hsa-let-7a-5p | Forward | Convergent | TGAGGTAGTAGGTTGTATAGTT |
|  | Reverse |  | AACTATACAACCTACTACCTCA |
| hsa-let-7b-5p | Forward | Convergent | TGAGGTAGTAGGTTGTGTGGTT |
|  | Reverse |  | AACCACACAACCTACTACCTCA |
| hsa-let-7c-5p | Forward | Convergent | TGAGGTAGTAGGTTGTATGGTT |
|  | Reverse |  | AACCATACAACCTACTACCTCA |
| hsa-let-7d-5p | Forward | Convergent | AGAGGTAGTAGGTTGCATAGTT |
|  | Reverse |  | AACTATGCAACCTACTACCTCT |
| hsa-let-7f-5p | Forward | Convergent | TGAGGTAGTAGATTGTATAGTT |
|  | Reverse |  | AACTATACAATCTACTACCTCA |
| hsa-let-7g-5p | Forward | Convergent | TGAGGTAGTAGTTTGTACAGTT |
|  | Reverse |  | AACTGTACAAACTACTACCTCA |
| circZNF609 | Forward | Convergent | TCCACACCGCTCAAATGTTA |
|  | Reverse |  | ATCCAGGATGGTCGTTTCAA |
| Dusp9 | Forward | Convergent | GCTACCTGGCCTACTACCTCC |
|  | Reverse |  | CATCAGAGCAGTCGGAGCCC |
| Ldlr | Forward | Convergent | TGACTCAGACGAACAAGGCTG |
|  | Reverse |  | ATCTAGGCAATCTCGGTCTCC |
| Phlda1 | Forward | Convergent | GAAGATGGCCCATTCAAAAGCG |
|  | Reverse |  | GAGGAGGCTAACACGCAGG |
| GAPDH | Forward | Divergent (back-to-back) | TGCCATGTAGACCCCTTGAA |
|  | Reverse |  | GGCTGCCCATTCATTTCCTT |

* The species of genes was *Homo sapiens*.

* “back-to-back” represents divergent primers designed following the back-to-back rule, and “sjod” represents splice junction overlapping divergent primers.

*The direction of primer sequences was 5’ to 3’.

**Table S3 siRNAs and sgRNAs**

| **Target Gene** | **RNA** | **Sequence** |
| --- | --- | --- |
| si-NC | siRNA | UUCUCCGAACGUGUCACGUTT |
| hsa_circ_0060927 | siRNA | GCCUCAGGGAAGGGGAAGACUTT |
| hsa_circ_0003563 | siRNA | CCUCGGGAACCCAGAAGUGGUTT |
| hsa_circ_0026782 | sgRNA target Jo | ATCTTGTGGAAAGGACGAAA |
|  | sgRNA target Sq2 | TCTTGTGGAAAGGACGAAAC |
|  | sgRNA target Jb | GAGGTCCCACTATGTTGCAC |

*The direction of the sequences is 5’ to 3’.

* Jo is the upstream Alu element of hsa_circ_0026782, and Sq2 and Jb are downstream Alu elements of hsa_circ_0026782.

**Table S4 Antibodies**

| **Target Gene** | **Product Code** | **Application** | **Dilution Ratio** | **Brand Company** |
| --- | --- | --- | --- | --- |
| Flag | HT201 | WB | 1:5000 | TransGen |
| CREB1 | ET1601-15 | RIP | 4μg | HuaBio |
|  |  | WB | 1:500 |  |
|  |  | IF | 1:50 |  |
| IMPDH1 | 22092-1-AP | RIP | 4μg | Proteintech |
|  |  | WB | 1:2500 |  |
| AGO2 | BM4920 | RIP | 4μg | BOSTER |
|  |  | WB | 1:1000 |  |
| m^6^A | Bes5203-2(S) | MeRIP | 4μg | BersinBio |
|  | 68055-1-Ig | dot blot | 1:2000 | Proteintech |
| eIF4G2 | 67428-1-Ig | RIP | 4μg | Proteintech |
|  |  | WB | 1:10000 |  |
| p-CREB1 (Ser133) | AF5785 | WB | 1:1000 | Beyotime |
|  |  | IF | 1:50 |  |
| p-CREB1 (Ser142) | AF3188 | WB | 1:1000 | Affinity |
|  |  | IF | 1:500 |  |
| GAPDH | 2118S | WB | 1:1000 | Cell Signaling Technology |
| H3 | 14269T | WB | 1:1000 | Cell Signaling Technology |
| HRP-Conjugated Alpaca anti-Rabbit IgG | HA1031 | WB | 1:50000 | HuaBio |
| HRP Conjugated Goat anti-Mouse IgG | HA1006 | WB | 1:50000 | HuaBio |
| Multi-rAb CoraLite® Plus 488-Goat Anti-Rabbit Recombinant Secondary Antibody (H+L) | RGAR002 | IF | 1:500 | Proteintech |
| Multi-rAb CoraLite® Plus 594-Goat Anti-Rabbit Recombinant Secondary Antibody (H+L) | RGAR004 | IF | 1:500 | Proteintech |

* “WB” represents the western blot analysis, “IF” represents Immunofluorescence staining, “RIP” represents RNA binding protein immunoprecipitation assay, and “MeRIP” represents methylated RNA immunoprecipitation assay.

**Supplementary Methods**

**CircRNA High-throughput Sequencing**

Briefly speaking, total RNAs were extracted from three keloid tissues and three normal scar tissues using TRIzol reagent (Biosharp, Shanghai, China). Then we used DNase I to digest the DNA fragments remained in the total RNA sample. The ribosomal RNAs (rRNAs) were removed from the total RNA sample by using the TIANSeq rRNA Depletion Kit (H/M/R) (TIANGEN, Beijing, China). Then the RNase R (Sangon, Shanghai, China) reaction system was performed on the total RNA sample to digest the linear RNA components. The total RNA sample were fragmented at a specific temperature and ion concentration, followed by double strain complementary DNAs (cDNAs) synthesis (in second strain synthesis, dUTPs were used in place of dTTPs). The “A” bases were then added to the 3’ end cDNAs, and the two ends were ligated with adapters. The second cDNA strain containing dUTPs were digested using Uracil-DNA Glycosylase (UDG) enzyme (Sangon, Shanghai, China), followed by PCR amplification. After constructing cDNA library and circularizing the library products, the single strain cDNAs undergone rolling circle replication to form DNA nanoballs (DNBs). Finally, the high-throughput sequencing was performed on the DNBseq platform (BGI, Shenzhen, China).

The original data generated by high-throughput sequencing were called raw reads. First, we filtered out reads with low quality, adapter contamination, and high unknown base N content through SOAPnuke ^[1]^ (<https://github.com/BGI-flexlab/SOAPnuke>) software. The filtered data were called clean reads. The clean reads were then aligned to human reference genome (GRCh37/hg19) (<http://www.genome.ucsc.edu>) and the result was shown via Circos ^[2]^ (<http://mkweb.bcgsc.ca/circos>) software for further predictions and annotations. The circRNA prediction was performed by CIRI ^[3]^ (<https://sourceforge.net/projects/ciri/>) and find_circ ^[4]^ (<https://github.com/marvin-jens/find_circ>) software, and the results of the two software were merged for subsequent analysis.

**Differentially Expressed CircRNAs (DEcircs) Analysis**

The circRNA annotation was carried out on CircBase ^[5]^ ([http://www.CircBase.org/](http://www.circbase.org/)), a database which merged and unified data sets of circRNAs. In this study, the expression of circRNAs was calculated according to the number of back-spliced reads aligned to both ends of circRNA. CIRI and find_circ were used to predict the number of back-spliced reads, and the final number of back-spliced reads was the average of the two results. Back-spliced reads per million mapped reads (RPM) standardized the effect of sequencing depth, and was used to represent the circRNA expression level in this study. The differentially expressed circRNAs (DEcircs) between keloid and normal scar were screened out by DEGseq algorithm ^[6]^ (fold change ≥ 2 and adjusted *p*-value (*q*-value) ≤ 0.001 ^[7, 8]^) according to RPM.

**Enrichment Analysis**

Based on the Gene Ontology (GO) and Kyoto Encyclopedia of Genes and Genomes (KEGG) official classifications, we performed annotation and functional categorization of the genes associated with DEcircs, and conducted enrichment analysis via Bioconductor package, clusterProfiler 4.0 ^[9]^ (<https://www.bioconductor.org/packages/clusterProfiler>). The significant enriched genes were distinguished by a modified Fisher’s exact test, with *q* ≤ 0.01.

**Protein Coding Ability Prediction**

In this study, the ORF pipeline ([https://github.com/kadenerlab/ORF_pipeline](https://github.com/kadenerlab/cORF_pipeline)) and IRESfinder ^[10]^ (<https://github.com/xiaofengsong/IRESfinder>) softwares were used to predict the open reading frames (ORFs) and ribosome entry site (IRES) of circRNA, respectively. The ORF pipeline annotated the circRNAs using known transcripts to find ORFs that cross backsplices. IRESfinder was a prediction tool for IRES based on logit model. In IRESfinder, each circRNA sequence was divided into 174 nucleotides (nt) in size and the regions sliding in steps of 50 nt were sequentially predicted for the likelihood of IRES, and a scoring value was given. The region with the highest score was considered the IRES sequence of this circRNA.

**MiRNAs Interaction Net Prediction**

In this study, we used miRanda ^[11]^ (<http://www.microrna.org/microrna/getGeneForm.do>) and RNAhybrid ^[12]^ (<http://bibiserv.techfak.uni-bielefeld.de/rnahybrid>) softwares to predict the potencial bingding miRNAs of DEcircs. The interaction network was constructed by R language.

**References**

1. CHEN Y, CHEN Y, SHI C, HUANG Z, ZHANG Y, LI S, LI Y, YE J, YU C, LI Z, ZHANG X, WANG J, YANG H, FANG L, CHEN Q. SOAPnuke: a MapReduce acceleration-supported software for integrated quality control and preprocessing of high-throughput sequencing data [J]. Gigascience, 2018, 7(1): 1-6.

2. KRZYWINSKI M, SCHEIN J, BIROL I, CONNORS J, GASCOYNE R, HORSMAN D, JONES S J, MARRA M A. Circos: an information aesthetic for comparative genomics [J]. Genome Res, 2009, 19(9): 1639-45.

3. GAO Y, WANG J, ZHAO F. CIRI: an efficient and unbiased algorithm for de novo circular RNA identification [J]. Genome Biology, 2015, 16(1): 4.

4. MEMCZAK S, JENS M, ELEFSINIOTI A, TORTI F, KRUEGER J, RYBAK A, MAIER L, MACKOWIAK S D, GREGERSEN L H, MUNSCHAUER M, LOEWER A, ZIEBOLD U, LANDTHALER M, KOCKS C, LE NOBLE F, RAJEWSKY N. Circular RNAs are a large class of animal RNAs with regulatory potency [J]. Nature, 2013, 495(7441): 333-8.

5. GLAŽAR P, PAPAVASILEIOU P, RAJEWSKY N. circBase: a database for circular RNAs [J]. Rna, 2014, 20(11): 1666-70.

6. WANG L, FENG Z, WANG X, WANG X, ZHANG X. DEGseq: an R package for identifying differentially expressed genes from RNA-seq data [J]. Bioinformatics, 2010, 26(1): 136-8.

7. BENJAMINI Y, HOCHBERG Y. Controlling the False Discovery Rate: A Practical and Powerful Approach to Multiple Testing [J]. Journal of the Royal Statistical Society Series B (Methodological), 1995, 57(1): 289-300.

8. STOREY J D, TIBSHIRANI R. Statistical significance for genomewide studies [J]. Proc Natl Acad Sci U S A, 2003, 100(16): 9440-5.

9. WU T, HU E, XU S, CHEN M, GUO P, DAI Z, FENG T, ZHOU L, TANG W, ZHAN L, FU X, LIU S, BO X, YU G. clusterProfiler 4.0: A universal enrichment tool for interpreting omics data [J]. Innovation (Camb), 2021, 2(3): 100141.

10. ZHAO J, WU J, XU T, YANG Q, HE J, SONG X. IRESfinder: Identifying RNA internal ribosome entry site in eukaryotic cell using framed k-mer features [J]. J Genet Genomics, 2018, 45(7): 403-6.

11. RIFFO-CAMPOS Á L, RIQUELME I, BREBI-MIEVILLE P. Tools for Sequence-Based miRNA Target Prediction: What to Choose? [J]. Int J Mol Sci, 2016, 17(12).

12. KRüGER J, REHMSMEIER M. RNAhybrid: microRNA target prediction easy, fast and flexible [J]. Nucleic Acids Res, 2006, 34(Web Server issue): W451-4.
